# Emergent memory in cell signaling: Persistent adaptive dynamics in cascades can arise from the diversity of relaxation time-scales

**DOI:** 10.1038/s41598-018-31626-9

**Published:** 2018-09-05

**Authors:** Tanmay Mitra, Shakti N. Menon, Sitabhra Sinha

**Affiliations:** 1 The Institute of Mathematical Sciences, CIT Campus, Taramani, Chennai, 600113 India; 2 Homi Bhabha National Institute, Anushaktinagar, Mumbai, 400094 India

## Abstract

The mitogen-activated protein kinase (MAPK) signaling cascade, an evolutionarily conserved motif present in all eukaryotic cells, is involved in coordinating crucial cellular functions. While the asymptotic dynamical behavior of the pathway stimulated by a time-invariant signal is relatively well-understood, we show using a computational model that it exhibits a rich repertoire of transient adaptive responses to changes in stimuli. When the signal is switched on, the response is characterized by long-lived modulations in frequency as well as amplitude. On withdrawing the stimulus, the activity decays over long timescales, exhibiting reverberations characterized by repeated spiking in the activated MAPK concentration. The long-term persistence of such post-stimulus activity suggests that the cascade retains memory of the signal for a significant duration following its removal. The molecular mechanism underlying the reverberatory activity is related to the existence of distinct relaxation rates for the different cascade components. This results in the imbalance of fluxes between different layers of the cascade, with the reuse of activated kinases as enzymes when they are released from sequestration in complexes. The persistent adaptive response, indicative of a cellular “short-term” memory, suggests that this ubiquitous signaling pathway plays an even more central role in information processing by eukaryotic cells.

## Introduction

Intra-cellular signaling networks are paradigmatic of complex adaptive systems that exhibit a rich repertoire of responses to stimuli^1^. Such networks mediate the response of a cell to a wide variety of extra- and intra-cellular signals, primarily through a sequence of enzyme-substrate biochemical reactions^2,3^. While the complexity of the entire signaling system is daunting^4^, it is possible to gain an insight into how it functions by focusing on a key set of frequently occurring motifs. These often take the form of linear signaling cascades, referred to as pathways. One of the best known of these pathways is the mitogen-activated protein kinase (MAPK) cascade that is present in all eukaryotic cells^5,6^. It is involved in regulating a range of vital cellular functions, including proliferation and apoptosis^6^, stress response^7^ and gene expression^8^. This signaling module comprises a sequential arrangement of three protein kinases, viz., MAPK, MAPK kinase (MAP2K) and MAPK kinase kinase (MAP3K). Modular function is initiated when extracellular signals stimulate membrane-bound receptors upstream of the cascade, with the information being relayed to MAP3K by a series of intermediaries. Activated kinases in each layer of the module function as enzymes for phosphorylating (and thereby activating) the kinase in the level immediately downstream, with the subsequent deactivation being mediated by corresponding dephosphorylating enzymes known as phosphatases (PPase). The terminal kinase in this cascade, i.e., MAPK, transmits the signal further downstream by phosphorylating various proteins, including transcription regulators^9^. Extensive investigations into the asymptotic dynamical behavior of the cascade have contributed towards an in-depth understanding of several emergent features including ultrasensitivity^10^, and oscillations^11,12^ that arise through retrograde propagation of activity^13–15^ or explicit feedback^16^. One of the striking features of the cascade is the occurrence of bistability, which allows the system to switch between two possible states corresponding to low and high activity^12,17–20^. This provides a post-transcriptional mechanism for obtaining a sustained response from transient signals, i.e., cellular memory^21,22^.

Memory can be understood as long-term alterations in the state of a system in response to environmental changes, which allow the system to retain information about transient signals long after being exposed to them^21^. This can arise in the cell through mechanisms such as auto-regulatory transcriptional positive feedback^23^ and nucleosomal modifications^24^. In the context of cell-fate determination, it has been shown that an irreversible biochemical response can be generated from a short-lived stimulus through feedback-based bistability^22^. This corresponds to a permanent alteration of the state of the system, thereby actively maintaining ‘memory’ of the signal. As bistability has also been observed to arise through multi-site phosphorylation in signaling modules, protein phosphorylation has been suggested as a plausible post-transcriptional mechanism for cellular memory^21,25,26^. In particular, there have been extensive investigations of the MAPK cascade as it integrates a large range of signals received by the cell in order to control numerous cellular decisions^27–33^. While majority of these investigations have considered the asymptotic dynamical behavior of the system, one may also observe transitory modulations in the response of the cascade in a changing environment^34,35^. The latter could encode information about prior stimuli to which the system was exposed, and can be a potential mechanism for imparting a form of “short-term” memory to the signaling cascade.

In this paper we show that a linear MAPK cascade can indeed exhibit short-term memory through transient modulations in its response to an environmental change. Crucially, this can arise even in the absence of explicit feedback between different layers or cross-talk with other pathways. These modulations can persist long after the initial trigger, lasting for durations that are several orders of magnitude longer than the time-scales associated with phosphorylation-dephosphorylation processes. We demonstrate that this occurs both when a signal begins activating the MAPK cascade, as well as when it is withdrawn. On application of the stimulus, the module exhibits long-lived frequency and amplitude modulations in the activation profile of the constituent kinases. Following the withdrawal of stimulus, activity in the cascade decays over an extremely long time-scale, during which reverberatory dynamics, characterized by large-amplitude spiking in MAP Kinase activity, can be observed. We explain the emergence of such long-lived memory of the withdrawn stimulus in terms of the imbalance of fluxes between different layers of the cascade, which results from the diversity of relaxation time-scales of the cascade components, and the reuse of activated kinases as enzymes when they are released from sequestration. This phenomenon is seen to be robust with respect to variations in the model parameters, including the kinetic rate constants and the molecular concentrations of the constituent kinases and phosphatases. Our results reveal that a biochemical signaling module as simple as the MAPK cascade is capable of exhibiting short-term memory that is manifested as persistent modulations in the adaptive response of the system to changes in stimuli.

## Results

**Figure 1:**
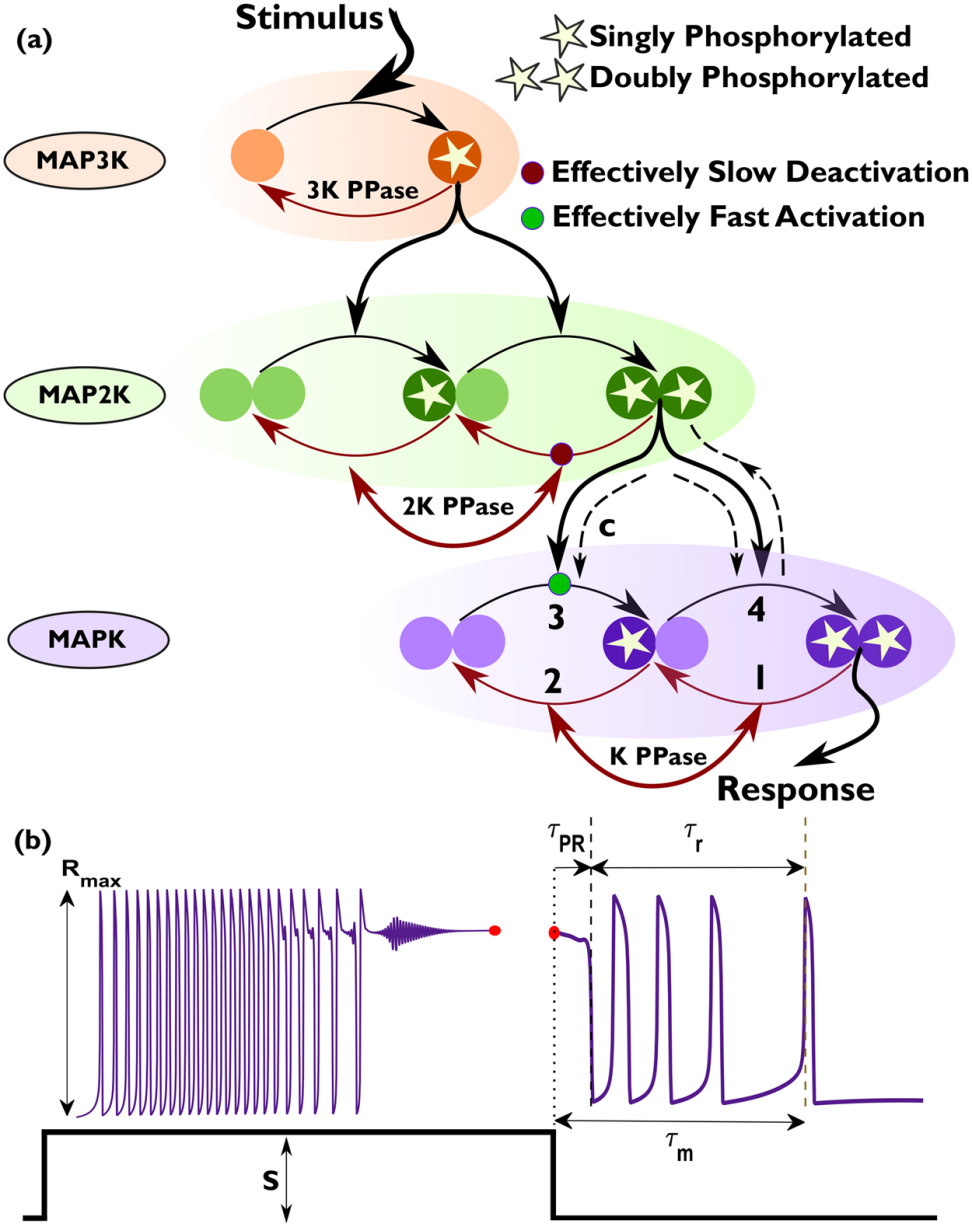
Adaptive response of MAPK cascade to a changing stimulus. (**a**) Schematic representation of a linear MAPK cascade comprising three layers. Signaling is initiated by a stimulus S activating MAPK kinase kinase (MAP3K). Activation/deactivation of kinases is achieved by adding/removing phosphate groups, which is referred to as phosphorylation/dephosphorylation respectively. The activated MAP3K regulates the phosphorylation of MAPK kinase (MAP2K). Doubly phosphorylated MAP2K, in its turn, controls the activation of MAPK. The response of the cascade to the signal is measured in terms of MAPK activity, viz., the concentration of doubly phosphorylated MAPK. Deactivation of a phosphorylated kinase is regulated by the corresponding phosphatase (indicated by PPase) in the corresponding layer of the cascade. The numbers 1–4 represent the sequence of events that lead to the emergence of a large amplitude spiking response following the withdrawal of the stimulus. The enzyme-substrate protein complex formed during activation of MAPK by doubly phosphorylated MAP2K is indicated by “c”. Broken lines have been used to highlight the principal processes that drive the reverberatory dynamics, which functions as a memory of the signal (see text for details). (**b**) Schematic illustrating the emergence of long-lived transient modulations of MAPK activity in response to initiation of a signal of optimal strength *S*. Withdrawing the stimulus can result in persistent large-amplitude spiking in the response of MAPK, suggestive of a form of “short-term” memory. The maximum response of MAPK to the stimulus is denoted by *R*_max_. The primary recovery time (*τ*_PR_) is characterized as the duration following withdrawal of stimulus after which MAPK activity decreases to its half-maximum value (*R*_max_/2) for the first time. The duration over which reverberatory dynamics occurs is indicated by *τ*_*r*_, while the total duration for which memory of the withdrawn stimulus persists is *τ*_*m*_ = *τ*_*PR*_ + *τ*_*r*_.

For the results reported in this paper we consider the Huang-Ferrell model of the MAPK signaling cascade^10^, schematically shown in Fig. 1(a). Typically, investigations into the dynamics of this model focus on the asymptotic response to sustained stimulation. For all the parameter sets used in this study, the system exhibits a characteristic sequence of transitions in its asymptotic dynamical state upon increasing the strength of the stimulus (see Supplementary Information). For the parameter sets used in our study, there is a lower critical value of the stimulus strength that demarcates a steady state regime characterized by low levels of MAPK activation from a large-amplitude oscillatory regime. Beyond an upper critical value of the stimulus strength, the oscillatory regime gives way to another steady state regime marked by high levels of MAPK activation. In contrast to such asymptotic dynamics, here we report on the transient activity of the system responding to a change in the stimulus. Specifically, we describe the response immediately following the introduction of a signal of amplitude *S* and that following its removal.

### Emergence of persistent modulations in kinase activity

**Figure 2:**
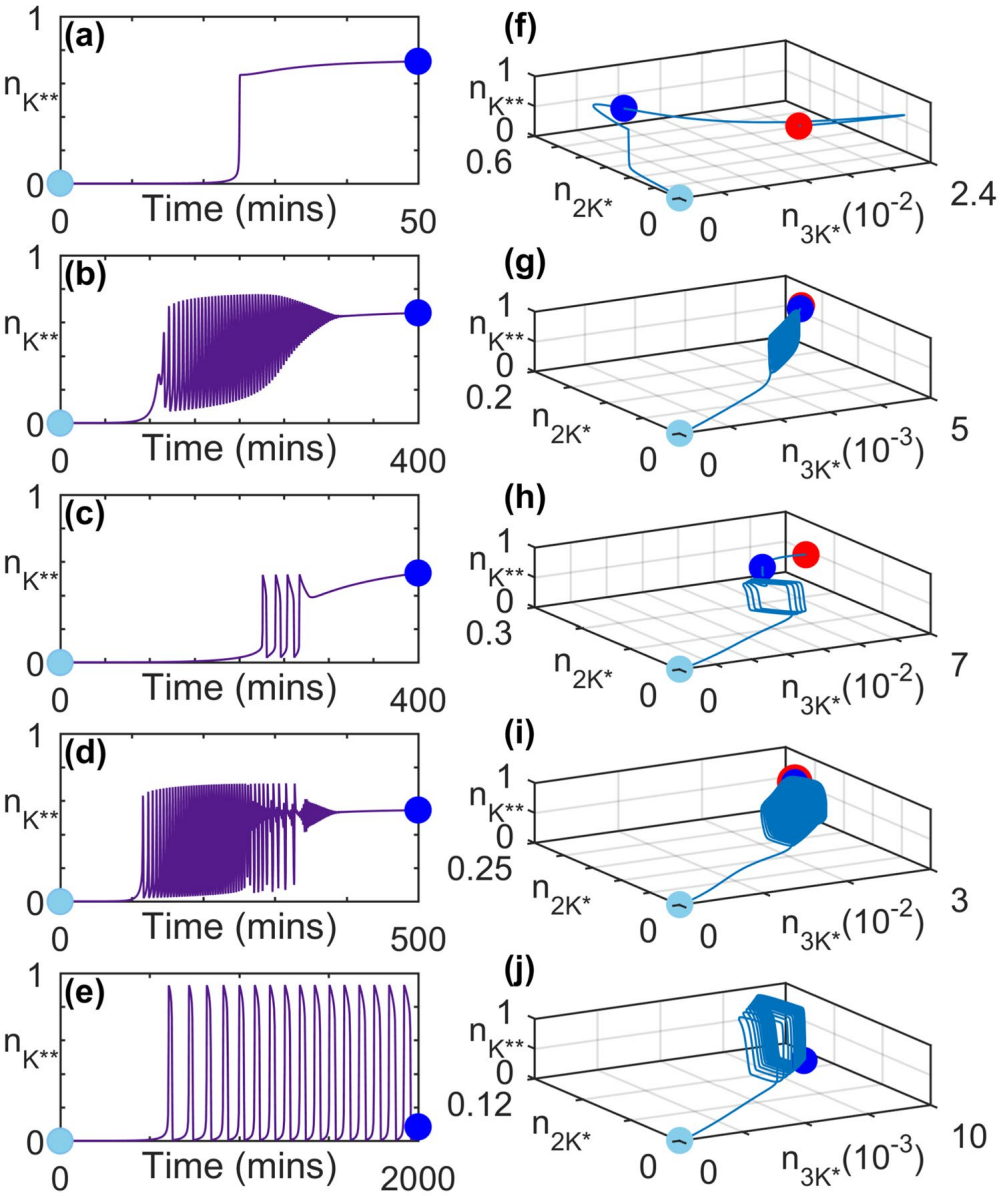
Transient activity in MAPK cascade immediately following the application of a stimulus at *t* = 0. (**a**–**e**) Characteristic time series for the normalized concentration of doubly phosphorylated MAPK (${n}_{{K}^{\ast \ast }}$), shown for different total concentrations of kinases. (**f**–**j**) Trajectories representing the evolution of the systems in panels (a–e) in the projection of the phase-space on the planes comprising normalized concentrations of active MAP3K (${n}_{3{K}^{\ast }}$), singly phosphorylated MAP2K (${n}_{2{K}^{\ast }}$) and active MAPK (${n}_{{K}^{\ast \ast }}$). The concentrations have been normalized by the total concentration of MAP3K ([3*K*]_*tot*_), MAP2K ([2*K*]_*tot*_) and MAPK ([*K*]_*tot*_), respectively. The light blue and dark blue markers in each of the panels (f–j) demarcate the portion of the trajectories that correspond to the time series shown in panels (a–e). The steady state of the system is represented by a red marker in panels (f–i). In panels (e) and (j), the system converges to a stable limit cycle. For details of parameter values and signal amplitudes for the systems shown in each of the panels see Table 1.

**Figure 3:**
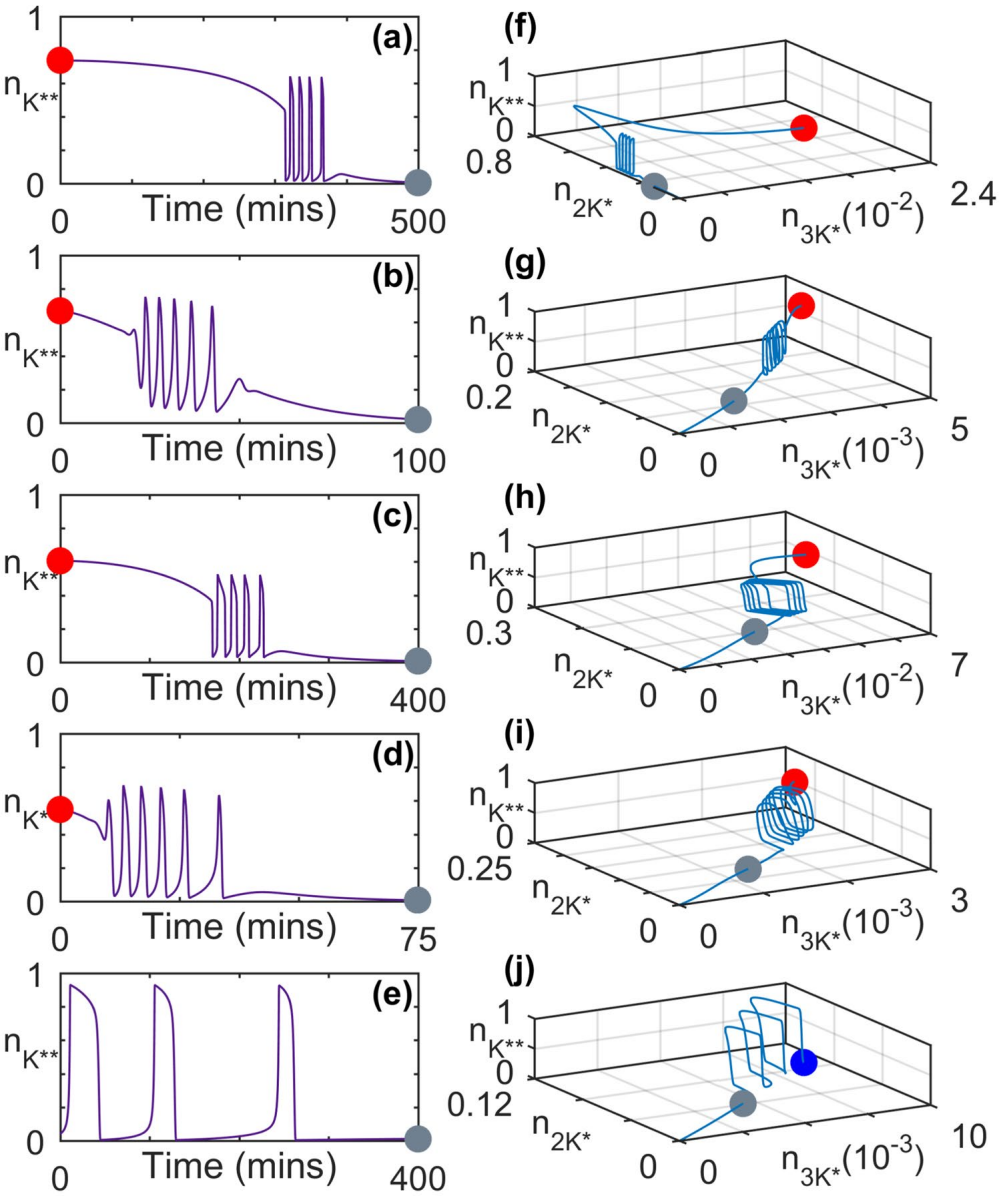
Transient activity in MAPK cascade immediately following the withdrawal (at *t* = 0) of an applied stimulus. (**a**–**e**) Characteristic time series for the normalized concentration of doubly phosphorylated MAPK (${n}_{{K}^{\ast \ast }}$) shown for different total concentrations of kinases. (**f**–**j**) Trajectories representing the evolution of the systems in panels (a–e) in the projection of the phase-space on the planes comprising normalized concentrations of active MAP3K (${n}_{3{K}^{\ast }}$), singly phosphorylated MAPK (${n}_{2{K}^{\ast }}$) and active MAPK (${n}_{{K}^{\ast \ast }}$). The concentrations have been normalized by the total concentration of MAP3K ([3*K*]_*tot*_), MAP2K ([2*K*]_*tot*_) and MAPK ([*K*]_*tot*_), respectively. The steady state of the system prior to the withdrawal of the stimulus is represented by a red marker (panels f–i). The system in panels (e) and (j) is seen to relax from a state characterized by stable limit cycle oscillations (represented by the blue marker). In each trajectory shown in (f–j) the grey marker denotes the state of the system corresponding to the final time point in panels (a–e). The concentration of active MAPK is close to its resting state value following the time period shown in (a–e). The parameter values and signal amplitudes for each panel are same as those for the corresponding panels in Fig. 2.

Our results reveal that the transient dynamics can be unexpectedly long-lived, lasting for durations that are much longer compared to the time-scales associated with the phosphorylation and dephosphorylation processes in the cascade (Fig. 1b). While most of the detailed results reported here were obtained using a set of parameter values that differ only marginally from the base values used by Huang & Ferrell^10^, qualitatively similar behavior can be observed for many other parameter sets drawn from a physiologically plausible range, as explicitly shown in Figs 2 and 3 (see Methods and Supplementary Information for a discussion on robustness).

**Table 1:** Signal amplitudes and system parameters for the panels in Figs 2 and 3.

Parameter	(a), (f)	(b), (g)	(c), (h)	(d), (i)	(e), (j)	Units
[*S*]	1.00	1.00	5.00	1.00	1.20	10^−6^ *μM*
[*K*]_*tot*_	2.43	4.51	2.66	3.10	3.20	*μM*
[2*K*]_*tot*_	3.96	4.64	2.87	1.63	1.80	*μM*
[3*K*]_*tot*_	1.26	1.48	0.32	1.25	0.24	10^−2^ *μM*
[*P*_*K*_]	4.88	2.75	5.88	1.75	0.50	10^−1^ *μM*
[*P*_2*K*_]	0.30	1.40	0.80	1.30	0.30	10^−3^ *μM*
[*P*_3*K*_]	1.96	3.72	2.70	1.02	1.00	10^−4^ *μM*
*k*_1_	1.40	1.24	1.47	3.67	1.00	10^3^ (*μM*.min)^−1^
*k*_−1_	1.84	2.32	4.91	3.68	1.50	10^2^ min^−1^
*k*_2_	6.28	2.32	4.01	7.26	1.50	10^2^ min^−1^
*kp*_1_	1.35	2.76	2.98	1.97	1.00	10^3^ (*μM*.min)^−1^
*kp*_−1_	4.71	5.60	1.67	6.76	1.50	10^2^ min^−1^
*kp*_2_	1.94	0.71	0.49	3.14	1.50	10^2^ min^−1^
*k*_3_	4.52	2.37	2.21	2.71	1.00	10^3^(*μM*.min)^−1^
*k*_−3_	3.07	3.92	6.80	2.82	0.30	10^2^ min^−1^
*k*_4_	6.46	4.14	2.66	1.53	0.30	10^2^ min^−1^
*kp*_3_	0.92	0.69	2.61	2.96	1.00	10^3^ (*μM*.min)^−1^
*kp*_−3_	5.86	2.90	6.37	1.81	1.50	10^2^ min^−1^
*kp*_4_	1.97	6.72	5.20	3.59	1.50	10^2^ min^−1^
*k*_5_	4.44	1.84	4.55	2.83	1.00	10^3^ (*μM*.min)^−1^
*k*_−5_	1.23	4.47	2.75	5.79	0.30	10^2^ min^−1^
*k*_6_	6.12	6.07	1.10	1.97	0.30	10^2^ min^−1^
*kp*_5_	3.55	2.08	2.26	4.82	1.00	10^3^ (*μM*.min)^−1^
*kp*_−5_	1.38	6.76	2.23	7.35	1.50	10^2^ min^−1^
*kp*_6_	7.09	5.52	3.17	6.82	1.50	10^2^ min^−1^
*k*_7_	4.41	4.28	2.70	4.69	1.00	10^3^ (*μM*.min)^−1^
*k*_−7_	1.70	2.59	5.24	2.35	0.30	10^2^ min^−1^
*k*_8_	2.06	3.42	1.38	1.27	0.30	10^2^ min^−1^
*kp*_7_	1.03	2.13	3.25	2.46	1.00	10^3^ (*μM*.min)^−1^
*kp*_−7_	2.76	1.28	4.84	1.18	1.50	10^2^ min^−1^
*kp*_8_	4.39	6.17	7.14	5.00	1.50	10^2^ min^−1^
*k*_9_	4.56	1.15	2.87	1.66	1.00	10^3^ (*μM*.min)^−1^
*k*_−9_	6.30	2.36	2.66	5.56	1.50	10^2^ min^−1^
*k*_10_	5.29	7.01	5.08	5.14	1.50	10^2^ min^−1^
*kp*_9_	2.72	8.14	1.86	3.39	1.00	10^3^ (*μM*.min)^−1^
*kp*_−9_	3.04	7.26	0.49	6.39	1.50	10^2^ min^−1^
*kp*_10_	2.23	3.97	3.57	6.49	1.50	10^2^ min^−1^

We first report the behavior of a cascade that is initially in the resting state (characterized by the absence of any phosphorylated components) when it is exposed to a signal. The transient activity that immediately follows exhibits several non-trivial features such as regular spiking in the activity of MAP2K and MAPK depending on the total concentrations of the kinases (Fig. 2b–e) and the signal strength. For a fixed initial state and signal strength, the spikes can furthermore show modulation in their frequency (Fig. 2c–e) as well as amplitude (Fig. 2b and d). In certain cases, both types of modulation can be observed (Fig. 2d). In the representative time series of MAPK activity shown in Fig. 2(a–e), the system dynamics eventually converges to a stable fixed point (Fig. 2a–d) or a stable limit cycle (Fig. 2e). Corresponding phase space projections are shown in Fig. 2(f–i). The complex modulations seen in many of these figures can arise as a result of coexisting attractors. For example, in Fig. 2(d) the system state appears to spend a considerable time in the basin of attraction of a limit cycle before approaching a stable fixed point (see Supplementary Information for details). Note that if the cascade components are already phosphorylated to an extent when the stimulus is switched on, the system will reach the asymptotic state much more rapidly thereby reducing the duration of transient activity.

An even more intriguing set of complex modulations is observed in the response of the cascade when the signal is withdrawn any time after the stimulated system has converged to the corresponding asymptotic state (which can be as short as a few minutes). Specifically, on switching off the signal, the cascade exhibits large-amplitude spiking behavior in the MAPK activity before eventually relaxing to the resting state (Fig. 3). The duration of the spiking activity and the inter-spike intervals can have a wide variety of time-scales as shown in Fig. 3(a–e). Corresponding phase space projections of the dynamics are shown in Fig. 3(f–i). This post-stimulus reverberatory activity is seen over a range of stimuli strengths and is indicative of a form of memory that can be achieved without explicit feedback or inter-pathway crosstalk. An essential condition for observing this phenomenon is that prior to withdrawing the applied stimulus, the system should have reached an asymptotic state corresponding to either large-amplitude oscillations or a steady state characterized by high MAPK activity. While the reverberatory activity shown in the different panels of Fig. 3 persist over durations ranging from less than an hour to a few hours, even longer periods of reverberation can be obtained depending on system parameters (see Supplementary Information).

### Processes underlying long-lived memory and reverberatory dynamics

**Figure 4:**
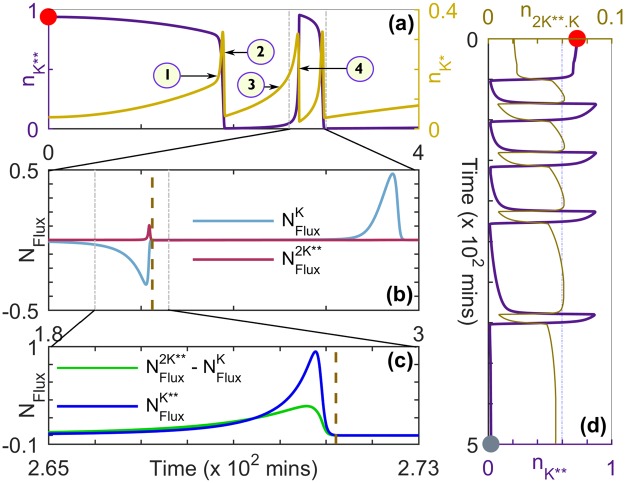
Processes underlying emergent memory and reverberatory dynamics in the MAPK cascade. (**a**) A characteristic time-series for the normalized concentrations of singly and doubly phosphorylated MAPK (${n}_{{K}^{\ast }}$ and ${n}_{{K}^{\ast \ast }}$, respectively) following the removal of an applied stimulus of amplitude *S* = 2.0 × 10^−6^ *μM* at *t* = 0. The numbers (1–4) represent the sequence of events that lead to the emergence of the post-stimulus large-amplitude spiking activity shown schematically in Fig. 1(b). (**b**) Normalized chemical flux *N*_Flux_ of MAPK and MAP2K** shown for the segment of the time-series where the spiking behavior in ${n}_{{K}^{\ast \ast }}$ is observed following the withdrawal of the stimulus to MAP3K [demarcated by broken vertical lines in (a)]. (**c**) Normalized chemical flux *N*_Flux_ of MAPK** shown along with the difference between the normalized fluxes of MAP2K** and MAPK for the duration indicated by broken vertical lines in (b) corresponding to the peak in the spiking activity of MAPK**. For both panels (b) and (c), normalization of flux is with respect to the maximum of the flux for MAPK**. (**d**) Characteristic time-series for the reverberatory activity of MAPK following the withdrawal of a stimulus of amplitude *S* = 1.2 × 10^−6^ *μM* at *t* = 0, showing the normalized concentration of MAPK** (${n}_{{K}^{\ast \ast }}$) along with that of the protein complex MAP2K**.MAPK (${n}_{2{K}^{\ast \ast }\mathrm{.}K}$ = [MAP2K**.MAPK]/[2*K*]_*tot*_). The reference line shows that the peak normalized concentration of the protein complex eventually decreases over time. For details of parameter values see Supplementary Information. The steady state of the system prior to the withdrawal of the stimulus is represented by a red marker [panels (a) and (d)], while the grey marker in (d) corresponds the final time point in Fig. 3(d).

When the stimulus is withdrawn from the MAPK cascade, the decline in MAP Kinase activity comes about through MAPK** binding to MAPK PPase which dephosphorylates it, resulting in an increased concentration of MAPK* [Step 1, Figs 1(a) and 4(a)]. In turn, the phosphatase binds to MAPK* thereby deactivating it to MAPK, which results in an extremely rapid decline in the concentration of MAPK* (Step 2). Concurrently, the deactivation of MAP2K** is delayed, as most of it is bound in the complex MAP2K**.MAPK that has a long time-scale of disassociation. To proceed further we can analyze the constituent processes in terms of the normalized chemical flux *N*_Flux_ of a molecular species, i.e., its rate of growth expressed relative to the maximum rate of growth of MAPK**. We observe that the suppression of MAP2K** deactivation mentioned above results in its normalized chemical flux exceeding that of MAPK [Fig. 4(b)]. Thus, there is a net growth in activity in the MAP Kinase layer as whenever MAP2K** is released from the complex, it is available to phosphorylate MAPK which results in an increase in the concentration of MAPK* (Step 3). The resulting rise in MAPK* manifests as a spike in its concentration [Fig. 4(a)], and it subsequently gets phosphorylated again to increase MAPK** concentration even in the absence of any stimulation (Step 4). When the net difference between the normalized flux of MAP2K** and MAPK reaches a maximum, the normalized chemical flux of MAPK** attains its highest value and consequently peak activity of MAP Kinase is observed [Fig. 4(c)]. Thus, steps 1–4 represent one complete cycle of MAP Kinase reverberatory activity characterized by an initial decline and a subsequent rise in MAPK** concentration. These steps are subsequently repeated a number of times resulting in a series of spikes in MAPK activity [Fig. 4(d)]. The abrupt nature of the rise and fall of MAP Kinase activity that manifests as spikes is a consequence of the bistable nature of the dynamics in the MAPK layer of the cascade^17,19^. In other words, MAP2K** can be bound either to the corresponding phosphatase (resulting in its subsequent deactivation) or to MAPK/MAPK* (which protects it from deactivation by being inaccessible to its phosphatase). This competition between the phosphatase and the downstream kinase results in a part of the available MAP2K** being sequestered for long times and thus being available for activating MAPK (on being released from the complex) long after the withdrawal of the original stimulus. This results in the post-stimulus repeated spiking activity in the MAPK reported here. We note that similar spiking behavior is also observed in the activity of MAP2K, with the phase of the MAP2K** spikes shifted slightly forward with respect to the corresponding ones in MAPK**, which suggests that they result from retrograde propagation of activity from the MAPK to the MAP2K layer^14^. On the other hand, MAP3K shows a monotonic decline in its activity following the removal of the stimulus.

**Figure 5:**
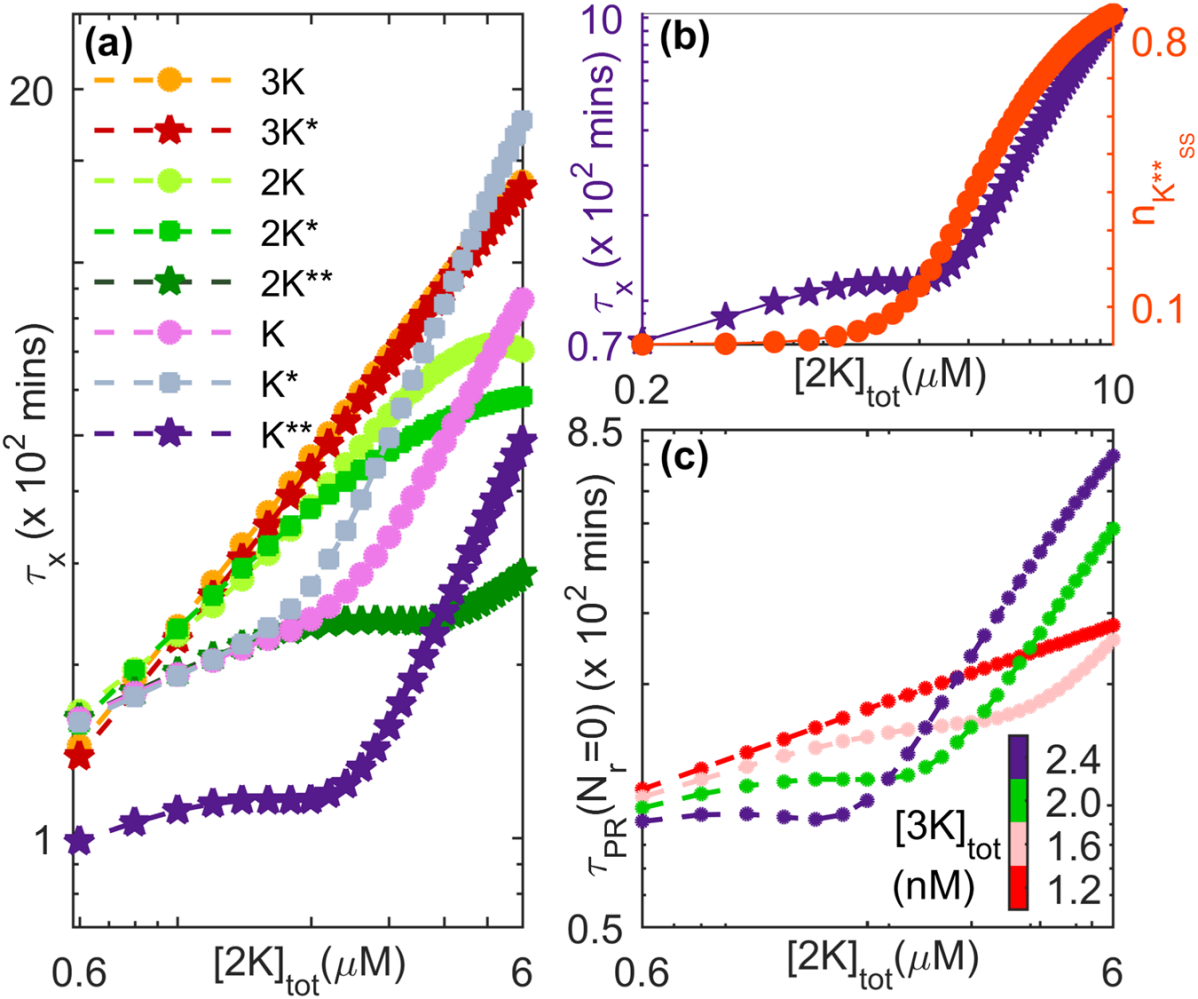
Components of the MAPK cascade exhibit relaxation behavior occurring over a broad range of time-scales. Decay of activity is shown after withdrawing an applied stimulus of amplitude *S* = 1.2 × 10^−6^ *μM*. (**a**) The relaxation times *τ*_x_ of the different molecular species (non, singly and doubly phosphorylated kinase proteins) in each of the layers of the cascade vary with the total concentration of MAP2K. The nature of this dependence is distinct for lower and higher values of [2*K*]_*tot*_, which is most prominently observed in the lower layers of the cascade. (**b**) The occurrence of distinct regimes in the relaxation behavior of MAPK** for different [2*K*]_*tot*_ is related to the corresponding increase in the steady state value attained by MAPK** concentration under sustained stimulation of the cascade. At a specific value of the steady-state normalized MAPK activity ${n}_{{K}^{\ast \ast }}$, we observe a crossover from the regime characterized by slowly increasing *τ*_x_ seen at lower total concentrations of MAP2K to a regime where *τ*_x_ increases relatively rapidly for higher [2*K*]_*tot*_. (**c**) The crossover behavior is also observed in the dependence of the closely related measure *τ*_*PR*_, the primary recovery time (see Methods), on [2*K*]_*tot*_. The difference between the two regimes become more prominent upon increasing the total concentration of MAP3K ([3*K*]_*tot*_). For both panels (a) and (b) [*K*]_*tot*_ = 0.8 *μM* and [3*K*]_*tot*_ = 2.0 *nM*, while for panel (c), [*K*]_*tot*_ = 0.8 *μM*. For details of all other parameter values see Supplementary Information.

**Figure 6:**
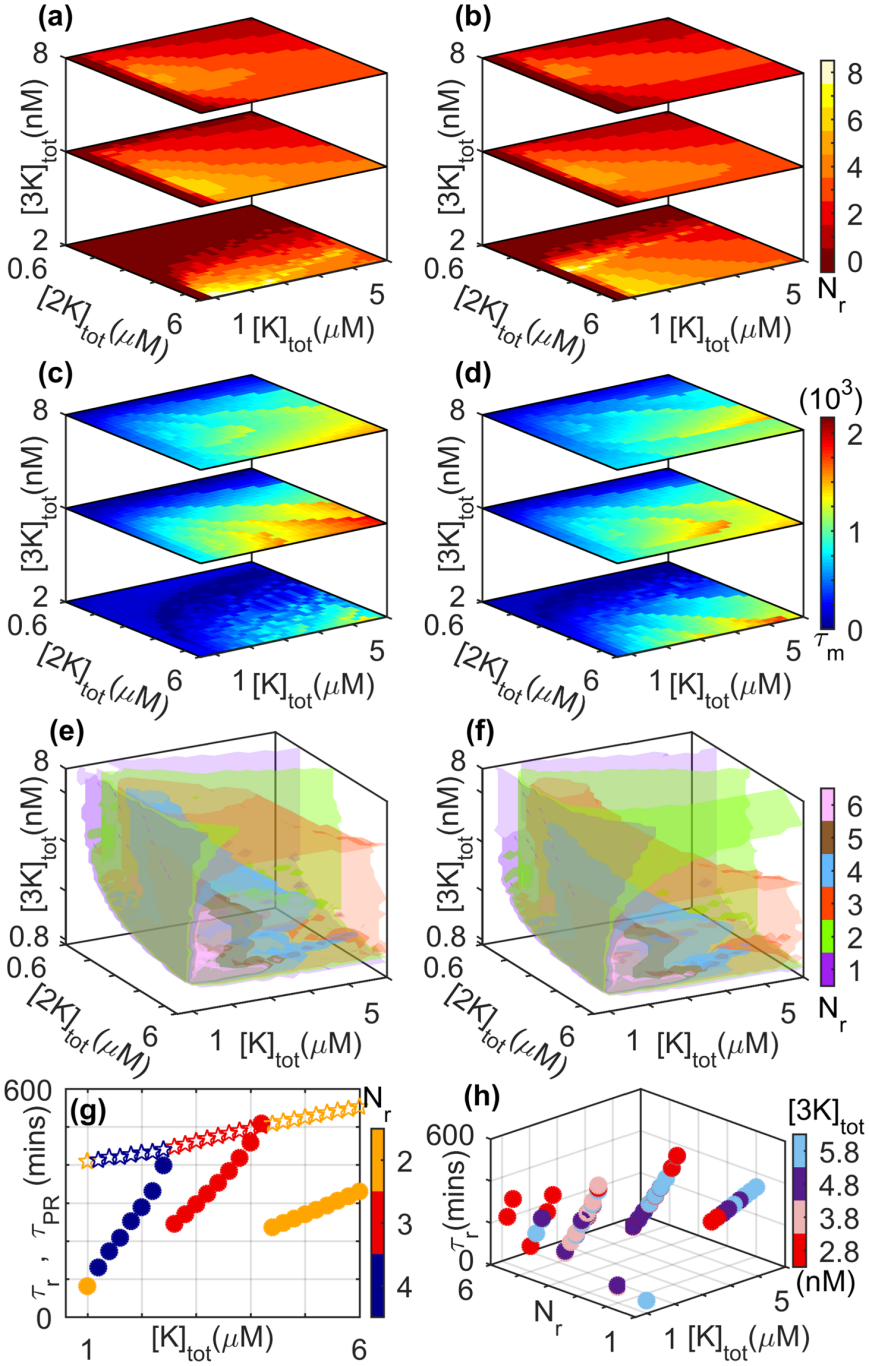
Dependence of reverberatory activity on the total kinase concentrations, viz., [*K*]_*tot*_, [2*K*]_*tot*_ and [3*K*]_*tot*_. (**a**,**b**) The number of spikes *N*_*r*_, (**c**,**d**) the total memory time *τ*_*m*_ (in minutes) and (**e**,**f**) isosurfaces for *N*_*r*_ observed on withdrawing an applied stimulus of amplitude *S* [=0.8 × 10^−6^ *μM* for (a,c,e) and 1.2 × 10^−6^ *μM* for (b,d,f)] are shown as functions of total concentrations of the three kinases. (**g**) The primary recovery time *τ*_PR_ (stars) and the total duration of reverberatory activity *τ*_*r*_ (filled circles) are shown for different values of *N*_*r*_ (indicated by the color bar). While *τ*_PR_ increases monotonically with increasing total MAPK concentration, *τ*_*r*_ shows a more complex dependence ([2*K*]_*tot*_ = 3 *μM* and [3*K*]_*tot*_ = 4 *nM*). (**h**) The dependence of *τ*_*r*_ on [*K*]_*tot*_ for different values of *N*_*r*_ has a similar nature for different choices of [3*K*]_*tot*_ (indicated by the color bar, [2*K*]_*tot*_ = 3 *μM*). Note that for panel (h), we consider only situations where the system attains a steady state on maintaining stimulation. For details of all other parameter values see Supplementary Information.

**Figure 7:**
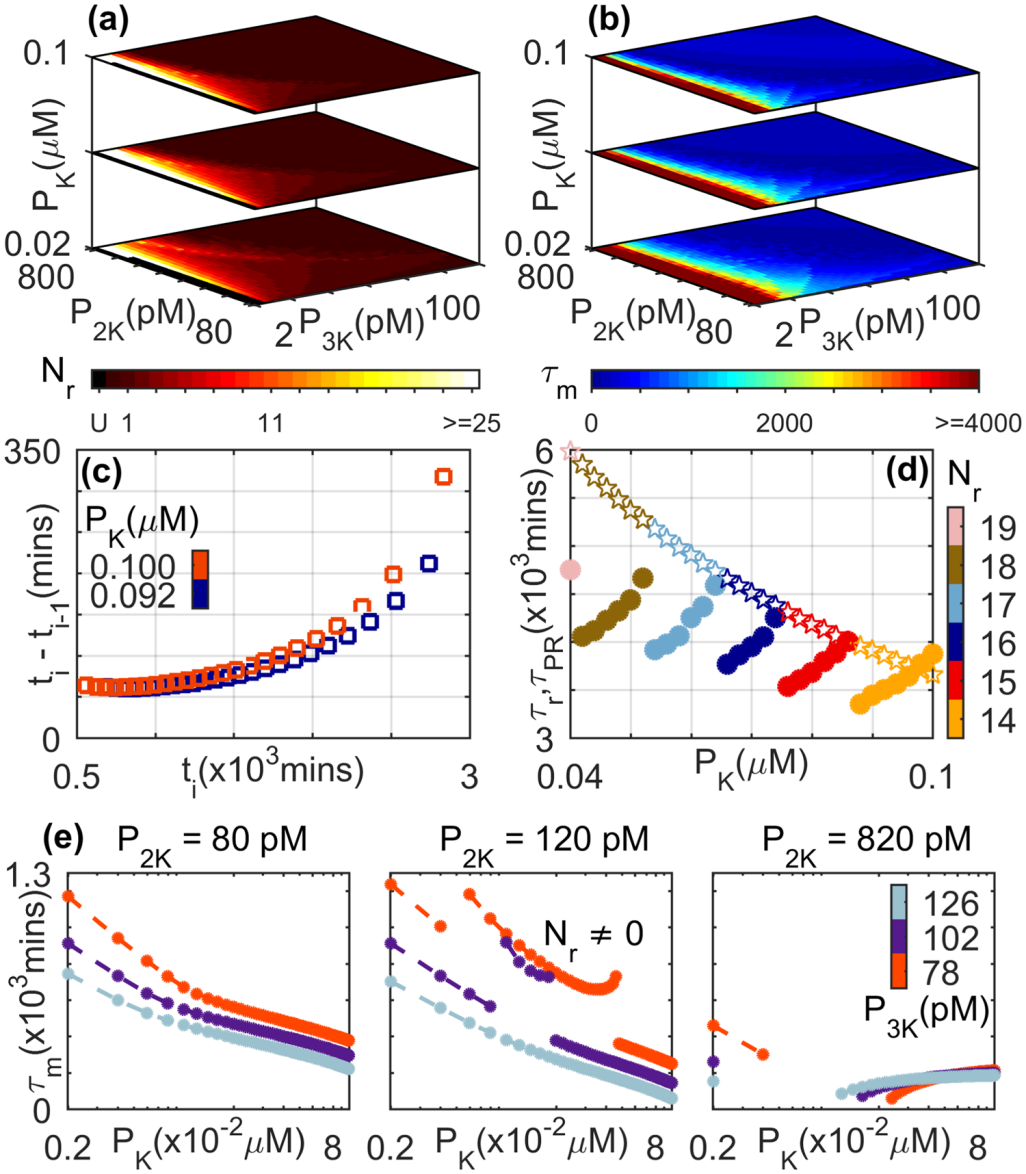
Dependence of reverberatory activity on the total concentrations of the phosphatases MAPK PPase ([*P*_*K*_]), MAP2K PPase, ([*P*_2*K*_]) and MAP3K PPase ([*P*_3*K*_]). (**a**) The number of spikes *N*_*r*_ and (**b**) the total memory time *τ*_*m*_ (in minutes) observed on withdrawing an applied stimulus of amplitude *S* = 0.8 × 10^−6^ *μM*. Situations where the primary recovery time is longer than a maximum or cut-off value (see Methods), such that the duration of the reverberatory dynamics cannot be properly measured, are indicated by the color corresponding to “U”. (**c**) The interval between successive spikes *i* − 1 and *i* increases with time (*t*_*i*_ being the time of occurrence of the *i* th spike). As the MAPK PPase concentration is increased, the durations of these intervals are seen to increase. The total concentrations of the other two phosphatases are maintained at [*P*_2*K*_] = 680 *pM* and [*P*_3*K*_] = 10 *pM*. (**d**) The variation of primary recovery time *τ*_PR_ (stars) and the total duration of reverberatory activity *τ*_*r*_ (filled circles) as a function of total MAPK PPase concentration are shown for different values of *N*_*r*_ (indicated by the color bar). While *τ*_PR_ decreases monotonically with increasing [*P*_*K*_], *τ*_*r*_ shows a more complex dependence ([*P*_2*K*_] = 200 *pM* and [*P*_3*K*_] = 6 *pM*). (**e**) Dependence of the total memory time *τ*_*m*_ on total MAPK PPase concentration ([*P*_*K*_] shown in log scale) for different total concentrations of MAP2K PPase (values indicated above each of the three panels) and MAP3K PPase (indicated using different colors as shown in the color bar). Note that we consider only situations where the system attains a steady state on maintaining stimulation. For details of all other parameter values see Supplementary Information.

In order to characterize in detail the memory of prior activity retained by the cascade which is manifested as long-lived transient reverberations following the withdrawal of stimulus, we use the following measures (see Methods): (i) the primary recovery time (*τ*_PR_), (ii) the number of spikes (*N*_*r*_) that occur during the relaxation process, (iii) the temporal intervals between successive spikes (*t*_*i*_−*t*_*i*−1_, where *t*_*i*_ is the time of occurrence of the *i* th spike event) and (iv) the total duration of reverberatory activity (*τ*_*r*_) following primary recovery. The total memory time (*τ*_*m*_) is the sum of *τ*_PR_ and *τ*_*r*_ as indicated in Fig. 1 (b). In the following we use these measures to present a detailed characterization of the behavior of the cascade components over a range of parameter values (Figs 5–7).

### MAP Kinase cascade components have different recovery timescales

As mentioned earlier, the emergence of long-lived reverberatory activity of MAPK following the withdrawal of an applied stimulus can be linked to the flux imbalance of different cascade components, which suggests significant differences in their rates of relaxation. As shown in Fig. 5(a), this is indeed the case, even for parameter regimes where no spiking activity of MAPK is observed (i.e., *N*_*r*_ = 0). As can be seen, the nature of increase of the relaxation time with increasing total concentrations of kinase protein MAP2K is distinct for the different molecular species and also depends on the state of their phosphorylation. In the lower layers of the cascade, we also find a crossover between two regimes seen at lower and higher values of [2*K*]_*tot*_ respectively. These regimes are characterized by relatively slow and rapid increases (respectively) in the recovery times with increasing [2*K*]_*tot*_, and appear to be related to the steady-state value attained by MAPK activity upon sustained stimulation of the cascade for the corresponding value of [2*K*]_*tot*_ [Fig. 5(b)]. The crossover between the two regimes is seen to occur for a value of [2*K*]_*tot*_ for which ~17% of MAPK is activated for the parameter values used in Fig. 5(b).

The distinct regimes are also observed in the dependence of the primary recovery time *τ*_PR_ on [2*K*]_*tot*_ [Fig. 5(c)]. As can be observed, the difference between the regimes becomes more pronounced with an increase in the total concentration of MAP3K. An important point to note is that for lower values of [2*K*]_*tot*_, the recovery time decreases with increasing [3*K*]_*tot*_ while the reverse trend is seen for higher values of [2*K*]_*tot*_. We have verified that increasing the stimulus amplitude *S* while keeping the total MAP3K concentration fixed has a similar effect on the relaxation behavior of activated MAPK (see Supplementary Information). As increasing total concentration of MAP2K results in increased steady-state activity of MAPK, we conclude that, in general, higher activity states of MAPK are associated with increasing relaxation time when either the signal or the substrate (MAP3K) is increased. Conversely, for states characterized by much lower MAPK activity, larger values of *S* or [3*K*]_*tot*_ results in reduced relaxation periods.

### Dependence of reverberatory activity on total kinase concentrations

Diverse cellular environments are characterized by different total concentrations of the various molecular components of the MAPK cascade. Thus, in order to determine the robustness of spiking and reverberatory activity following the removal of an applied stimulus, it is important to see how they are affected by varying total kinase concentrations. Such a study will also indicate the ease with which these phenomena can be experimentally observed. Figure 6 shows the variation of different measures of reverberatory activity on the total concentrations of MAPK, MAP2K and MAP3K. While there is a complex dependence on these parameters for the exact number of spikes *N*_*r*_ and the duration of the total memory time *τ*_*m*_, the phenomenon of reverberatory activity following withdrawal of stimulation can be observed over a large range of the parameter space, underlining its robustness. We also observe that on increasing [3*K*]_*tot*_, the response of *N*_*r*_ to variation in [*K*]_*tot*_ and [2*K*]_*tot*_ becomes relatively homogeneous. Increasing the stimulus amplitude *S* [compare panels (a,c,e) with (b,d,f) of Fig. 6] does not seem to alter the qualitative nature of the variation in *N*_*r*_ and *τ*_*m*_ over the parameter space in general, although we do observe that the domains corresponding to different values of *N*_*r*_ occupy different regions [Fig. 6(e and f)]. Note that for low [3*K*]_*tot*_, high values of *N*_*r*_ are observed to coexist with low values of *τ*_*m*_ [Fig. 6(a,c and b,d)]. While it may appear surprising that these two measures of memory are not in consonance in this region of parameter space, it can be explained by noting that the stimulated system is in an oscillatory state, and following the removal of the signal these relatively high-frequency oscillations cease after a short duration. Figure 6(g) suggests that the variation seen in *τ*_*m*_ as a function of the total MAPK concentration for a specific *N*_*r*_ is mostly governed by *τ*_*r*_, the total duration of reverberatory activity, with the corresponding dependence of *τ*_*PR*_ on [*K*]_*tot*_ being weak.

As the total MAPK concentration is increased, we observe that while the primary recovery time increases almost linearly, the nature of the reverberatory dynamics as reflected in *τ*_*r*_ shows a more complex dependence on [*K*]_*tot*_ [Fig. 6(g)]. If for a given value of [*K*]_*tot*_ the MAPK activity following withdrawal of the stimulus shows *N*_*r*_ spikes over a duration of *τ*_*r*_, then on increasing [*K*]_*tot*_ the time-interval between the spikes increases (thereby resulting in an increase of *τ*_*r*_) until a critical value beyond which the last of the *N*_*r*_ spike no longer appears. Thus, at this point *N*_*r*_ reduces by unity with a concomitant drop in *τ*_*r*_. This series of events is repeated for steadily decreasing values of *N*_*r*_ as the total MAPK concentration is increased further. Each value of *N*_*r*_ is associated with a characteristic rate of increase in *τ*_*r*_ with [*K*]_*tot*_. With a reduction in *N*_*r*_ (as a result of increasing [*K*]_*tot*_), this rate is found to decrease as well, which suggests a saturation of the system response. These results are robust with respect to different choices of total MAP3K concentration as can be seen from Fig. 6(h), suggesting that similar behavior will be seen for a range of strengths for the applied signal (see Supplementary Information).

### Dependence of reverberatory activity on total phosphatase concentrations

We have also investigated the role that phosphatase availability plays on the reverberatory activity of the cascade following the withdrawal of the stimulus. As is the case for total kinase concentrations shown in Fig. 6, we see from Fig. 7(a,b) that the number of spikes *N*_*r*_ and the duration of total memory time *τ*_*m*_ depend on the total concentrations of the phosphatases MAPK PPase, MAP2K PPase, and MAP3K PPase. For larger values of the concentrations, viz., [*P*_*K*_], [*P*_2*K*_] and [*P*_3*K*_], respectively, the system operates in the low-amplitude response regime. As mentioned earlier, the reverberatory MAPK dynamics during recovery following withdrawal of the applied stimulus will not be seen in this regime. As the phosphatase concentrations are decreased, spiking behavior of MAPK activity is observed with both *τ*_*m*_ and *N*_*r*_ attaining high values in an optimal range. The large variation seen in *τ*_*m*_ [Fig. 7(b)] arises as regions in [*P*_2*K*_]-[*P*_3*K*_] parameter space characterized by the same value of *N*_*r*_ are seen to exhibit a range of different values of *τ*_*r*_ and *τ*_*PR*_ [Fig. 7(d)]. For reverberatory activity associated with a specific *N*_*r*_, we observe that the duration *τ*_*r*_ increases with increasing total MAPK PPase concentration. This is a consequence of the intervals between successive spikes (*t*_*i*_ − *t*_*i*−1_) increasing with [*P*_*K*_] as is shown in Fig. 7(c). Note that the results are qualitatively similar for different amplitudes of the applied stimulus (see Supplementary Information). However, increasing [*P*_*K*_] results also in decreased time for primary recovery *τ*_*PR*_ [Fig. 7(d)], which in conjunction with the previously mentioned result leads to non-monotonic dependence of the total memory time *τ*_*m*_ on phosphatase availability. While this non-monotonicity is suggested in Fig. 7(b), it is seen clearly in Fig. 7(e) where the central panel corresponds to situations where spiking behavior is observed in MAPK activity. Investigating the dependence of *τ*_*m*_ on *P*_*K*_ [Fig. 7(e)] reveals that the range of [*P*_*K*_] over which reverberatory activity (i.e., *N*_*r*_ ≠ 0) occurs is demarcated by discontinuities in the functional dependence of *τ*_*m*_ on *P*_*K*_. For intermediate *P*_2*K*_ [Fig. 7(e), central panel] where the system attains a steady state on maintaining stimulation, the spiking activity following withdrawal of the stimulus becomes more prominent for low total concentration of MAP3K PPase. For higher *P*_2*K*_ [Fig. 7(e), right panel] where the system becomes oscillatory over an intermediate range of [*P*_*K*_], reverberatory activity is observed over a broader range of [*P*_3*K*_]. While we have assumed that the same phosphatase acts on both the singly and doubly phosphorylated forms of the kinase in a particular layer of the cascade (as in the canonical Huang-Ferrell model), we have explicitly verified that our results are not sensitively dependent on this.

### Dependence of reverberatory activity on model parameters

**Figure 8:**
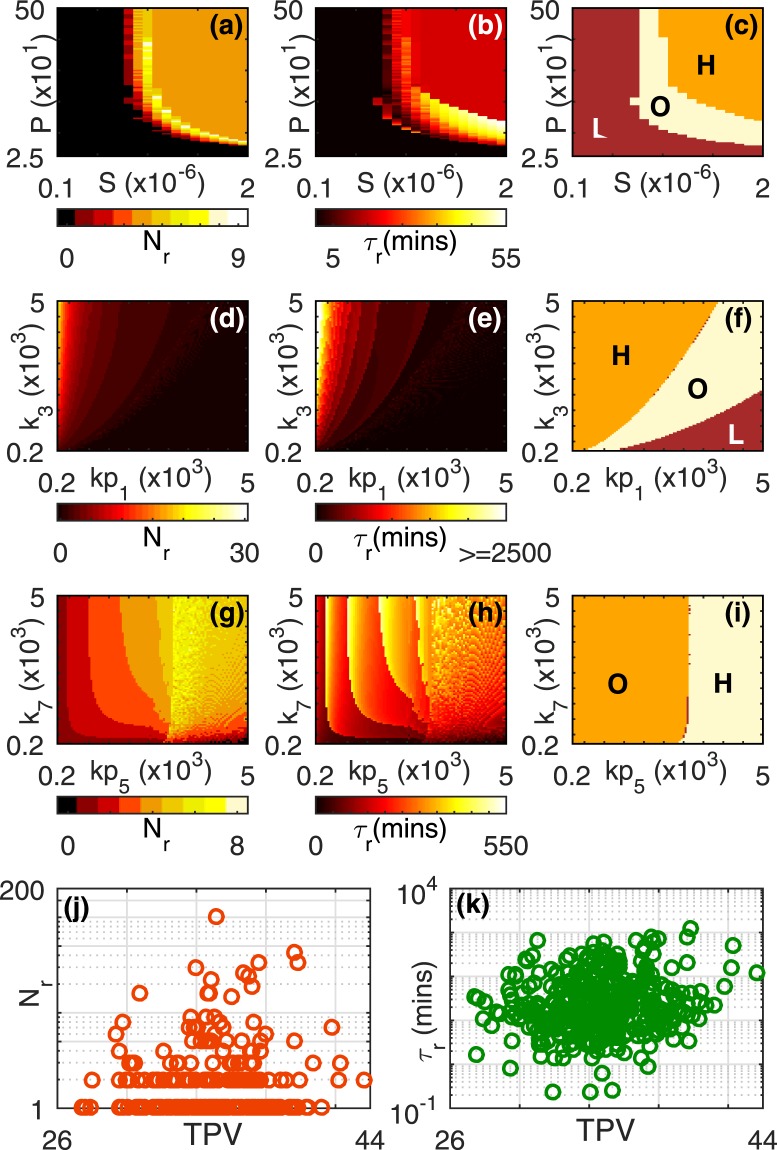
Dependence of the post-stimulus reverberatory activity of the MAPK cascade on extrinsic and intrinsic parameters. The corresponding dynamical attractors of the system under sustained stimulation are also shown. (**a**,**d**,**g**) The number of post-stimulus spikes *N*_*r*_ and (**b**,**e**,**h**) the total duration of reverberatory activity *τ*_*r*_ (in minutes) observed on withdrawing the stimulus, as well as, (**c**,**f**,**i**) the corresponding asymptotic dynamical states of the cascade under sustained stimulation, are shown. They are displayed as a function of (a–c) the stimulus strength *S* and its duration *P* (in mins), (d–f) the kinetic rates *kp*_1_ and *k*_3_ (both measured in *μM* min^−1^) which govern the enzyme-substrate complex formation steps in the dephosphorylation of MAP3K and single phosphorylation of MAP2K (respectively), and (g–i) the kinetic rates *kp*_5_ and *k*_7_ (both measured in *μM*. min^−1^) which govern the enzyme-substrate complex formation steps in the dephosphorylation of activated MAP2K and single phosphorylation of MAPK (respectively). The strength of the signal used to stimulate the cascade in all cases is *S* = 2 × 10^−6^ *μM*. For values of all other parameters see Table 1 [column corresponding to (e),(j)]. (**j**,**k**) Robustness of the observed reverberatory activity in MAPK cascade following withdrawal of applied stimulus having strength *S* = 10^−6^ *μM* with respect to variation in the system parameters. The panels show (j) the number of spikes during relaxation *N*_*r*_ and (k) the total duration of reverberatory activity *τ*_*r*_, on the Total Parameter Variation (TPV, as described in Methods). The circles in each panel represent an individual realization of the cascade dynamics where each parameter set is chosen by uniform random sampling from a physiologically plausible range (see Table S3 in Supplementary Information for details).

In addition to the effects of kinase and phosphatase concentrations considered above, we have studied how the reverberatory dynamics depends on the different extrinsic and intrinsic model parameters. Figure 8(a,b) shows how the number of spikes *N*_*r*_ and the total duration of reverberatory activity *τ*_*r*_ depend on the signal strength *S* and the time interval *P* for which it is applied prior to withdrawal. From Fig. 8(c) we observe that for large values of *S* and *P* the system operates in the high-amplitude response (H) regime. On lowering these stimulation parameters sufficiently a transition to an oscillatory dynamical regime (O) occurs. Further decrease in *S* and *P* results in yet another transition, this time to a low-amplitude response (L) regime. Note that, while reduction in the duration of stimulation can be compensated by increasing the signal strength in order to drive the system to the O or H regime starting from L, it appears that there is a critical value of *S* below which dynamical transitions cannot be achieved even if *P* is increased indefinitely.

As can be seen, the reverberatory MAPK dynamics following the withdrawal of the stimulus occurs only in the O and H regimes of the *S*–*P* parameter space. Intriguingly, the peak of reverberatory activity appears in the region corresponding to the boundary between these two dynamical regimes. This is analogous to *critical slowing down* in the response of physical systems which is manifested in the divergence of relaxation times near a phase transition^36^. However, there does not appear to be a simple relation between the asymptotic dynamical regimes observed on sustained stimulation of the cascade and the nature of the transient reverberatory activity, when we consider the dependence on intrinsic parameters such as the kinetic rate constants that govern the different reaction steps in the cascade. For example, in Fig. 8(d,e) we observe the variation of *N*_*r*_ and *τ*_*r*_ as a function of the rates *kp*_1_ and *k*_3_. As can be seen by comparing with the dynamical regimes in parameter space shown in Fig. 8(f), the peak of reverberatory activity occurs quite far from any of the transition zones. On the other hand, when we consider the variation of *N*_*r*_ and *τ*_*r*_ as a function of the rates *kp*_5_ and *k*_7_ [Fig. 8(g,h)], the peak of *N*_*r*_ does appear to coincide with the boundary between the O and H regimes [Fig. 8(i)], while the *τ*_*r*_ dependence is more complex, showing a number of local maxima. The dependence of the reverberatory activity on several other reaction rates of the system are shown in Supplementary Information.

Apart from looking at the role that individual parameters play in the post-stimulus activity of the cascade, we have also considered how simultaneous variation of all of the parameters affect the reverberatory dynamics. This allows us to investigate whether the phenomena are robust, an essential property if they are to be observed experimentally, as environmental variations, polymorphisms or mutations can often cause multiple parameters of the signaling cascade to be altered. We have verified that the reverberations are not sensitively dependent on system parameters by simulating the dynamics of the cascade using a large number of different parameter sets, each being obtained by randomly sampling the parameter values from their respective physiologically plausible ranges. Figure 8(j,k) shows that both in terms of the number of spikes *N*_*r*_, as well as, the total duration of reverberatory activity *τ*_*r*_, the phenomena of post-stimulus repeated spiking in the activated MAPK concentration we report here is not confined to a very small region of the parameter space but can be seen for a wide variety of choices for the parameter values. Thus, these results establish the robust nature of the emergent “short-term” cellular memory.

## Discussion

In this paper we have shown that an isolated MAPK signaling module can serve as a fundamental motif in the intra-cellular signaling network for imparting a form of short-term memory to the cell. The emergence of long-lived reverberatory activity reported here arises from the diversity of relaxation timescales for the different components of the MAP Kinase cascade, which results in flux imbalance between activation of the MAPK layer and deactivation in the MAP2K layer. One may therefore expect to observe results qualitatively similar to what has been reported here whenever the system has disparate timescales regardless of the actual molecular concentrations and kinetic rates which can vary substantially across different cells^37–39^. Thus, as the MAPK cascade is present in all eukaryotic cells^5,6^, the mechanism for short-term memory in such a signaling cascade that is presented here may hold for such cells in general. As the duration of MAPK** activity is critical for many cellular decisions^40^, e.g., the prolonged activation of ERK resulting in its translocation to the nucleus^41^, the persistent reverberatory activity seen here may play a non-trivial role in regulation of cellular functions. In addition, it has recently been shown that frequency modulation of ERK activity pulses can encode information controlling cellular proliferation^42^, suggesting that the pulsatile nature of the reverberatory MAPK activity shown here may need to be taken into account when considering such processes.

The basal level activity of MAPK in a normal cell is maintained at a low proportion of the total MAPK concentration and serves several biological functions^43^. We observe a crossover between two qualitatively distinct regimes of relaxation behavior of MAPK** occurring at a steady state that is characterized by relatively low proportion of activation of the available MAPK [~17% in Fig. 5(b)]. Thus, there appears to be an effective threshold for MAPK activity (which may be related to its basal state level) that demarcates the different relaxation regimes following the removal of the applied stimulus. A similar crossover is also observed for the primary recovery time *τ*_*PR*_.

One of the most challenging aspects of computational modeling of the dynamics of biological networks is correctly assigning the values of the large number of parameters associated with these models^44^. For the MAPK cascade investigated here, several of the kinetic rates associated with different reactions have never been measured experimentally. There are also a variety of values for the system parameters that have been reported in the literature^11^. For our investigations we have primarily used a reference set of parameter values (see SI for details) that differ only marginally from the base values originally used by Huang & Ferrell^10^, and yet which allow the cascade to exhibit three distinct dynamical regimes under different conditions. These are characterized by a time-invariant low-amplitude response (L), oscillatory dynamics (O) and a time-invariant high-amplitude response (H), respectively. We note that the phenomena we observe by using this reference parameter set in the cascade model is robust with respect to variations in the values of the parameters. Indeed, as reported above, the post-stimulus reverberatory activity of the cascade is observed for a large number of different parameter sets that have been randomly sampled from a physiologically plausible range.

It is known that ERK MAPK isoforms (e.g., p42 and p44) are abundantly expressed in non-dividing terminally differentiated neurons^28^. Activation of MAPK by spaced stimulation is known to be responsible for morphological changes in dendrites^27^. Studies also suggest that the activation of the MAPK pathway is linked with associative learning in the mammalian nervous system, synaptic plasticity and neurological memory^27,28,33,45,46^. An intriguing possibility suggested by the results reported here is that the observed repeated spiking in MAPK activity may function as an effective temporally spaced signal to the nucleus of a neuron. This can then facilitate subsequent changes in the cell required for memory formation.

Another well-known example of eukaryotic cellular memory is observed during chemotactic migration along the gradient of a chemical signal^47,48^. The directionality of migration is known to persist for a certain duration, even if the chemical gradient is altered or becomes static. Studies show that the protein Moesin contributes to the long-lived rigidity of the cytoskeleton assembly that subsequently leads to the directional memory in polarized migrating cells^48^. However, the intra-cellular processes that underlie the persistent activity of Moesin in the absence of a gradient mediated signal are still largely unknown. Evidence suggests that the regulation of Moesin and other ERM proteins are linked with the activity of the MAPK pathway^49,50^. The long-term reverberatory activity of MAPK following the withdrawal of a stimulus that is reported here may be a possible mechanism underlying such persistent cellular behavior.

To the best of our knowledge the post-stimulus reverberatory activity described here is yet to be reported in the experimental literature. This could possibly be because, typically, during experiments, recording of ERK activity is stopped soon after the withdrawal of the stimulus. Note that in all cases where we observe reverberations, it was preceded by an apparent monotonic decay of MAPK activity immediately following the withdrawal of the stimulus. Experiments would necessarily have to be carried out for much longer durations beyond this initial decay of MAPK activity (i.e., the primary recovery time *τ*_*PR*_) in order to observe the phenomenon reported here. It is of course possible that in the cellular milieu, coupling of the MAPK cascade to other intra-cellular signaling pathways, as well as, the possible presence of explicit feedback connections, may mask the response that is seen here in the case of an isolated MAPK cascade. Also, *in vivo* the cascade will be subject to a variety of signals that will often arrive in close succession. This will make it unlikely to observe extremely long-lived post-stimulus responses lasting over tens or hundreds of minutes. However, we hope that the results reported here will stimulate experiments specifically designed to test the existence of an emergent “short-term” memory in intra-cellular signaling.

To conclude, we have shown the possibility of long-lived reverberatory activity in a signaling cascade following the withdrawal of external stimuli. Our results suggest a mechanism through which the intra-cellular signaling system can encode short-term memory of signals to which the cell was previously exposed. The large-amplitude spiking activity of MAPK following the removal of a prior stimulus may also provide a mechanism for signal integration and learning when the cascade is repeatedly stimulated. We note that there may be additional factors not considered here that may lengthen the persistence of reverberatory activity, including scaffold proteins that increase the lifetime of kinase complexes. Our results suggest that the MAPK cascade potentially has a key role in shaping the information processing capabilities of eukaryotic cells in diverse environments.

## Methods

The dynamics of the three layer MAPK signaling cascade has been simulated using the Huang-Ferrell model^10^. Each of the constituent kinase and phosphatase-mediated enzyme-substrate reactions comprise (i) a reversible step corresponding to the formation of the enzyme-substrate complex and (ii) an irreversible product formation step corresponding to the activation/deactivation of a kinase, as described in the Supplementary Information.

The time-evolution of the molecular concentrations of the different components of the cascade are modeled using a set of coupled ordinary differential equations (see Supplementary Information) that are integrated using the stiff solver ode15s implemented in *MATLAB Release 2010b*. Note that the quasi-steady-state hypothesis has not been invoked^51^. To ensure that initially all kinases are non-phosphorylated we prepare the initial resting state of the system by simulating it for a long duration (~10^6^ mins) in the absence of any signal. Subsequently MAP3K is exposed to a stimulus of amplitude *S* and duration 5000 minutes. On using the base values for the parameter set as given in ref.^10^, the system exhibits ultrasensitivity (as reported earlier) which provides verification of the correct numerical implementation of the model (see Supplementary Information). Following the removal of the stimulus, we continue to simulate the system until it returns to the resting state or the simulation duration exceeds 10^4^ minutes.

We have analyzed the long-lived reverberatory activity of the cascade after the removal of the stimulus by using the following measures:

### The primary recovery time (*τ*_PR_)

Following the activation of the cascade by introducing a stimulus, the maximum concentration *R*_max_ of MAPK** is recorded. On removing the stimulus, MAPK activity starts to decay. The time taken for MAPK** to monotonically decrease to half of *R*_max_ is defined as the primary recovery time (*τ*_*PR*_).

### Number of spikes during relaxation (*N*_*r*_)

Following primary recovery, MAPK activity may exhibit a series of spikes, which are defined to be occurring whenever MAPK** concentration exceeds 70% of *R*_max_. The number of such spikes that are observed before the cascade reaches its resting state is designated as *N*_*r*_.

### The total duration of reverberatory activity (*τ*_*r*_)

When spiking is observed in MAPK activity following the removal of the applied stimulus, the reverberatory activity duration is defined as the interval between the termination of primary recovery and the final spike event, i.e., *τ*_*r*_ = *t*_*final*_ − *τ*_*PR*_. The time of the *i* th spike *t*_*i*_ is defined as the instant when MAPK activity reaches maximum during that particular event. For ${\tau }_{PR} > 6000$ mins, the total duration of the reverberatory activity may not be measured accurately as the total simulation duration does not exceed 10^4^ minutes.

### The total memory time (*τ*_*m*_)

The total duration of memory activity following removal of the applied stimulus is defined as the sum of the primary recovery time and the total duration of reverberatory activity, i.e., *τ*_*m*_ = *τ*_*PR*_ + *τ*_*r*_. Note that when the asymptotic dynamical behavior of the cascade in presence of the signal is oscillatory, on withdrawing the signal the activity may decay extremely rapidly resulting in *τ*_*m*_ ≈ 0.

### Relaxation time (*τ*_x_)

For the situations where the steady state corresponds to a fixed-point attractor we define a relaxation time *τ*_x_ for each constituent of the cascade. This is the time required by its concentration to evolve to the half-way point between the resting state and steady state values.

### Robustness Analysis

In order to investigate the robustness of the results reported here with respect to variations in the parameter values, we have performed simulations over an ensemble of cascade models whose parameter sets are obtained by uniform random sampling over a physiologically plausible range (see Supplementary Information). The deviation of such a randomly sampled parameter set from the base values used by Huang & Ferrell^10^ is measured by the Total Parameter Variation (TPV)^52^: $TPV={\sum }_{i=1}^{n}|{\mathrm{log}}_{10}({p}_{i}/{p}_{i,HF})|$, where *p*_*i*_ represents the value of the *i*-*th* parameter in the given sample and *p*_*i*,*HF*_ is the corresponding base value (*i* denotes any one of the 32 system parameters whose values are varied in this analysis). We have measured different characteristics of reverberatory activity, viz., *N*_*r*_, *τ*_*m*_ and *τ*_*r*_, for each realization of the cascade (corresponding to a particular random set of parameter values) and have observed them as a function of the TPV for four different values of the stimulus strength. We observe that qualitatively similar results to those reported here are observed for many different realizations.

## Electronic supplementary material

Supplementary Information

## Data Availability

The simulation data generated during the current study are available from the corresponding author on reasonable request.
