# Supplementary material for: Emergent memory in cell signaling: Persistent adaptive dynamics in cascades can arise from the diversity of relaxation time-scales
Source: Sci Rep. 2018 Sep 5;8:13230. doi: 10.1038/s41598-018-31626-9 (PMC6125488; doi:10.1038/s41598-018-31626-9)
Supplement: Supplementary file 1 — Supplementary Information [file 41598_2018_31626_MOESM1_ESM.pdf]

SUPPLEMENTARY INFORMATION
for
"Emergent memory in cell signaling: Persistent adaptive dynamics in cascades can arise from the diversity of relaxation time-scales "

Tanmay Mitra, Shakti N. Menon and Sitabhra Sinha

S1 The Model Equations

Table S1: Components of the MAPK Cascade

Component	Notation	Symbol
Mitogen-activated Protein Kinase Kinase Kinase	MAP3K	3K
Singly Phosphorylated Mitogen-activated Protein Kinase Kinase Kinase	MAP3K*	3K*
Mitogen-activated Protein Kinase Kinase	MAP2K	2K
Singly Phosphorylated Mitogen-activated Protein Kinase Kinase	MAP2K*	2K*
Doubly Phosphorylated Mitogen-activated Protein Kinase Kinase	MAP2K**	2K**
Mitogen-activated Protein Kinase	MAPK	K
Singly Phosphorylated Mitogen-activated Protein Kinase	MAPK*	K*
Doubly Phosphorylated Mitogen-activated Protein Kinase	MAPK**	K**
MAP3K-Phosphatase	3K PPase	P_{3K}
MAP2K-Phosphatase	2K PPase	P_{2K}
MAPK-Phosphatase	K PPase	P_{K}

The three layer MAPK cascade comprises the following enzyme-substrate reactions:

$$S + 3K \underset{k_{-1}}{\overset{k_1}{\rightleftharpoons}} S.3K \xrightarrow{k_2} S + 3K^*$$

$$P_{3K} + 3K^* \underset{kp_{-1}}{\overset{kp_1}{\rightleftharpoons}} 3K^*.P_{3K} \xrightarrow{kp_2} P_{3K} + 3K$$

$$3K^* + 2K \underset{k_{-3}}{\overset{k_3}{\rightleftharpoons}} 3K^*.2K \xrightarrow{k_4} 3K^* + 2K^*$$

$$P_{2K} + 2K^* \underset{kp_{-3}}{\overset{kp_3}{\rightleftarrows}} 2K^*.P_{2K} \xrightarrow{kp_4} P_{2K} + 2K$$

$$3K^* + 2K^* \underset{k_{-5}}{\overset{k_5}{\rightleftarrows}} 3K^*.2K^* \xrightarrow{k_6} 3K^* + 2K^{**}$$

$$P_{2K} + 2K^{**} \underset{kp_{-5}}{\overset{kp_5}{\rightleftarrows}} 2K^{**}.P_{2K} \xrightarrow{kp_6} P_{2K} + 2K^*$$

$$2K^{**} + K \underset{k_{-7}}{\overset{k_7}{\rightleftarrows}} 2K^{**}.K \xrightarrow{k_8} 2K^{**} + K^*$$

$$P_K + K^* \underset{kp_{-7}}{\overset{kp_7}{\rightleftarrows}} K^*.P_K \xrightarrow{kp_8} P_K + K$$

$$2K^{**} + K^* \underset{k_{-9}}{\overset{k_9}{\rightleftarrows}} 2K^{**}.K^* \xrightarrow{k_{10}} 2K^{**} + K^{**}$$

$$P_K + K^{**} \underset{kp_{-9}}{\overset{kp_9}{\rightleftarrows}} K^{**}.P_K \xrightarrow{kp_{10}} P_K + K^*$$

The above enzyme-substrate reactions can be expressed in terms of the following coupled ordinary differential equations (ODEs):

$$\begin{aligned}
\frac{d[3K]}{dt} &= k_{-1}.[S.3K] + kp_2.[3K^*.P_{3K}] - k_1.[S].[3K]\,, \\
\frac{d[S.3K]}{dt} &= k_1.[S].[3K] - (k_{-1} + k_2).[S.3K]\,, \\
\frac{d[3K^*.P_{3K}]}{dt} &= kp_1.[P^f_{3K}].[3K^*] - (kp_2 + kp_{-1}).[3K^*.P_{3K}]\,, \\
\frac{d[3K^*]}{dt} &= k_2.[S.3K] + kp_{-1}.[3K^*.P_{3K}] - kp_1.[P^f_{3K}].[3K^*] \\
&\quad +(k_{-3} + k_4).[3K^*.2K] - k_3.[3K^*].[2K] \\
&\quad +(k_{-5} + k_6).[3K^*.2K^*] - k_5.[3K^*].[2K^*]\,, \\
\frac{d[2K]}{dt} &= k_{-3}.[3K^*.2K] + kp_4.[2K^*.P_{2K}] - k_3.[3K^*].[2K]\,, \\
\frac{d[3K^*.2K]}{dt} &= k_3.[3K^*].[2K] - (k_{-3} + k_4).[3K^*.2K]\,, \\
\frac{d[2K^*.P_{2K}]}{dt} &= kp_3.[P^f_{2K}].[2K^*] - (kp_4 + kp_{-3}).[2K^*.P_{2K}]\,, \\
\frac{d[2K^*]}{dt} &= k_4.[3K^*.2K] + kp_{-3}.[2K^*.P_{2K}] - kp_3.[P^f_{2K}].[2K^*] \\
&\quad +k_{-5}.[3K^*.2K^*] - k_5.[3K^*].[2K^*] + kp_6.[2K^{**}.P_{2K}]\,,
\end{aligned}$$

$$
\begin{aligned}
\frac{d[3K^*.2K^*]}{dt} &= k_5.[3K^*].[2K^*] - (k_6 + k_{-5}).[3K^*.2K^*]\,, \\
\frac{d[2K^{**}.P_{2K}]}{dt} &= kp_5.[P^f_{2K}].[2K^{**}] - (kp_6 + kp_{-5}).[2K^{**}.P_{2K}]\,, \\
\frac{d[2K^{**}]}{dt} &= k_6.[3K^*.2K^*] + kp_{-5}.[2K^{**}.P_{2K}] - kp_5.[P^f_{2K}].[2K^{**}] \\
&\quad +(k_{-7} + k_8).[2K^{**}.K] - k_7.[2K^{**}].[K] \\
&\quad +(k_{-9} + k_{10}).[2K^{**}.K^*] - k_9.[2K^{**}].[K^*]\,, \\
\frac{d[K]}{dt} &= k_{-7}.[2K^{**}.K] + kp_8.[K^*.P_K] - k_7.[2K^{**}].[K]\,, \\
\frac{d[2K^{**}.K]}{dt} &= k_7.[2K^{**}].[K] - (k_8 + k_{-7}).[2K^{**}.K]\,, \\
\frac{d[K^*.P_K]}{dt} &= kp_7.[P^f_K].[K^*] - (kp_{-7} + kp_8).[K^*.P_K]\,, \\
\frac{d[K^*]}{dt} &= k_8.[2K^{**}.K] + kp_{-7}.[K^*.P_K] - kp_7.[P^f_K].[K^*] \\
&\quad +k_{-9}.[2K^{**}.K^*] - k_9.[2K^{**}].[K^*] + kp_{10}.[K^{**}.P_K]\,, \\
\frac{d[2K^{**}.K^*]}{dt} &= k_9.[2K^{**}].[K^*] - (k_{-9} + k_{10}).[2K^{**}.K^*]\,, \\
\frac{d[K^{**}.P_K]}{dt} &= kp_9.[P^f_K].[K^{**}] - (kp_{-9} + kp_{10}).[K^{**}.P_K]\,, \\
\frac{d[K^{**}]}{dt} &= k_{10}.[2K^{**}.K^*] + kp_{-9}.[K^{**}.P_K] - kp_9.[P^f_K].[K^{**}]\,.
\end{aligned}
$$

where

$$
\begin{aligned}
[S] &= [S]_{\text{tot}} - [S.3K]\,, \\
[P^f_{3K}] &= [P_{3K}] - [3K^*.P_{3K}]\,, \\
[P^f_{2K}] &= [P_{2K}] - [2K^*.P_{2K}] \; - [2K^{**}.P_{2K}]\,, \\
[P^f_K] &= [P_K] - [K^*.P_K] \; - [K^{**}.P_K]\,.
\end{aligned}
$$

Note that our simulations explicitly incorporate the constraint that the total concentrations of each type of kinase and phosphatase are conserved at all times. The concentrations of the different molecular species can vary over several orders of magnitudes. We have therefore numerically solved the equations using the stiff solver *ode15s* (implemented in *MATLAB Release 2010b*) with low relative and absolute tolerances in order to ensure the accuracy of the resulting time-series.

S2 System Parameters

The numerical values for the reaction rates used for most of the results reported here (viz., panels (e) & (j) of Fig. 2–3 and Figs. 4–7) have been obtained from Jesan *et al.* (2013) [cited in the main text]. A comparison between these values used in our study (MMS) and the base values of the parameters proposed by Huang & Ferrell (1996) [cited in the main text] is shown in Table S2. Note that the values for the kinetic rate constants used here differ only marginally from the base values (the deviating values are indicated in red).

Table S2: Reaction Rates

Rate constant	HF	MMS	Units
k_1	1000	1002	$(\mu M.\text{min})^{-1}$
k_{-1}	150	150	min^{-1}
k_2	150	150	min^{-1}
kp_1	1000	1002	$(\mu M.\text{min})^{-1}$
kp_{-1}	150	150	min^{-1}
kp_2	150	150	min^{-1}
k_3	1000	1002	$(\mu M.\text{min})^{-1}$
k_{-3}	150	30	min^{-1}
k_4	150	30	min^{-1}
kp_3	1000	1002	$(\mu M.\text{min})^{-1}$
kp_{-3}	150	150	min^{-1}
kp_4	150	150	min^{-1}
k_5	1000	1002	$(\mu M.\text{min})^{-1}$
k_{-5}	150	30	min^{-1}
k_6	150	30	min^{-1}
kp_5	1000	1002	$(\mu M.\text{min})^{-1}$
kp_{-5}	150	150	min^{-1}
kp_6	150	150	min^{-1}
k_7	1000	1002	$(\mu M.\text{min})^{-1}$
k_{-7}	150	30	min^{-1}
k_8	150	30	min^{-1}
kp_7	1000	1002	$(\mu M.\text{min})^{-1}$
kp_{-7}	150	150	min^{-1}
kp_8	150	150	min^{-1}
k_9	1000	1002	$(\mu M.\text{min})^{-1}$
k_{-9}	150	150	min^{-1}
k_{10}	150	150	min^{-1}
kp_9	1000	1002	$(\mu M.\text{min})^{-1}$
kp_{-9}	150	150	min^{-1}
kp_{10}	150	150	min^{-1}

The signal amplitudes and system parameters used to generate the representative time-series shown in all panels of Figs. 2 and 3 are listed in Table 1 of the main text. Note that the kinetic rates for panels (e, j) of Fig. 2 and Fig. 3 are same as the MMS values listed in Table S2.
In order to investigate the robustness of the results reported in the paper with respect to

variations in the parameter values, we have performed simulations over an ensemble of cascade models whose parameter sets are obtained by uniform random sampling over a physiologically plausible range given in Table S3. The random values thus obtained are further subject to the constraint that the resulting Michaelis-Menten constant K (defined as $K = (k_r + k_{cat})/k_f$, where k_f, k_r and k_{cat} are the forward, reverse and catalytic rate constants, respectively) should not exceed 1500 nM as per Huang & Ferrell (1996) [cited in main text].

Table S3: Biologically plausible range of the parameters used for random sampling

Parameter	Range of values	Units
$[K]_{tot}$	$0.075 - 6$	μM
$[2K]_{tot}$	$0.075 - 6$	μM
$[3K]_{tot}$	$1.875 \times 10^{-4} - 1.5 \times 10^{-2}$	μM
$[P_K]$	$7.5 \times 10^{-3} - 0.6$	μM
$[P_{2K}]$	$1.875 \times 10^{-5} - 1.5 \times 10^{-3}$	μM
$[P_{3K}]$	$1.875 \times 10^{-5} - 1.5 \times 10^{-3}$	μM
k_f	$62.5 - 5000$	$(\mu M.\text{min})^{-1}$
k_r	$9.36 - 750$	min^{-1}
k_{cat}	$9.36 - 750$	min^{-1}

Table S4: Total concentration (in μM) of the kinase proteins for Fig. 4 (panels a–c) and Fig. 7

$[K]_{tot}$	$[2K]_{tot}$	$[3K]_{tot}$
4.8	1.2	0.0030

Table S5: Total concentration (in μM) of the phosphatase proteins for Figs. 4–6 and Figs. S6-S7

$[P_{3K}]$	$[P_{2K}]$	$[P_K]$
1×10^{-4}	3×10^{-4}	0.05

Table S6: Total concentration (in μM) of the phosphatase proteins for Figs. S6 and S7

Panels	$[K]_{tot}$	$[2K]_{tot}$	$[3K]_{tot}$
(a) and (f)	3.0	3.0	0.0080
(b) and (g)	1.0	2.4	0.0024
(c) and (h)	1.2	6.0	0.0028
(d) and (i)	2.0	2.2	0.0024
(e) and (j)	4.8	6.0	0.0014

S3 Supplementary Figures

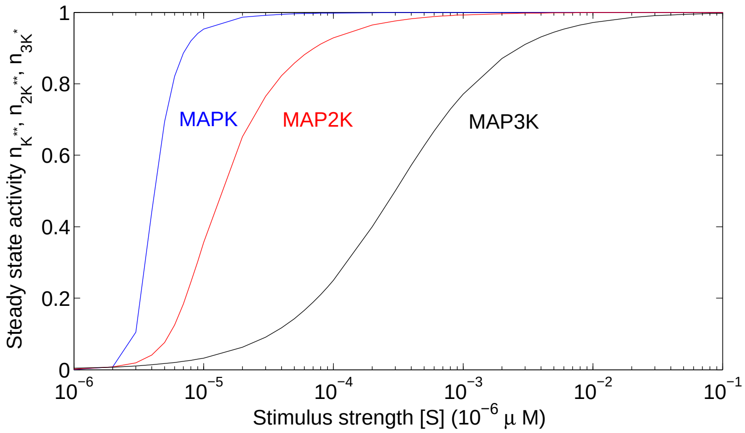

Figure S1: Steady-state kinase activity obtained in our numerical implementation of the MAPK cascade dynamics reproducing the results obtained by Huang & Ferrell (1996) [cited in the main text], using their base values for the parameters.

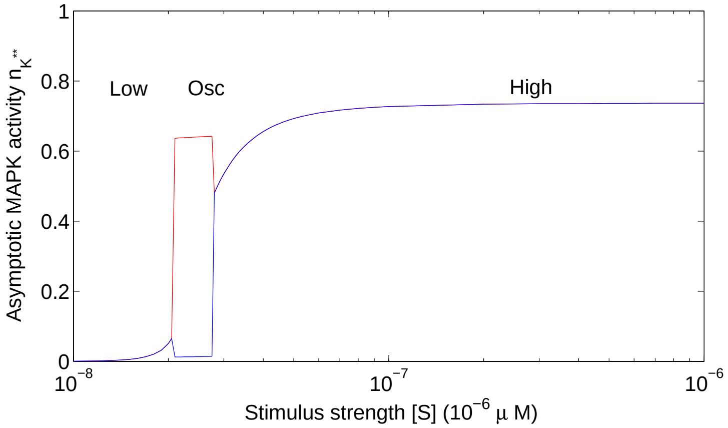

Figure S2: Representative bifurcation diagram of the MAPK cascade dynamics showing the asymptotic activity of MAPKinase as a function of the strength of the applied stimulus S. As the strength is increased, the behavior shows successive transitions from a low-response steady state regime ("Low") to large-amplitude oscillations ("Osc") and finally to a high-response steady state ("High") regime. The red and blue curves represent, respectively, the maxima and minima of MAPK activity in the oscillatory regime. The parameter set used is identical to that used for generating the time-series shown in Fig. 2 (a) [see Table 1 in the main text]. Qualitatively similar bifurcation diagrams are seen for all other parameter sets that give rise to reverberatory activity.

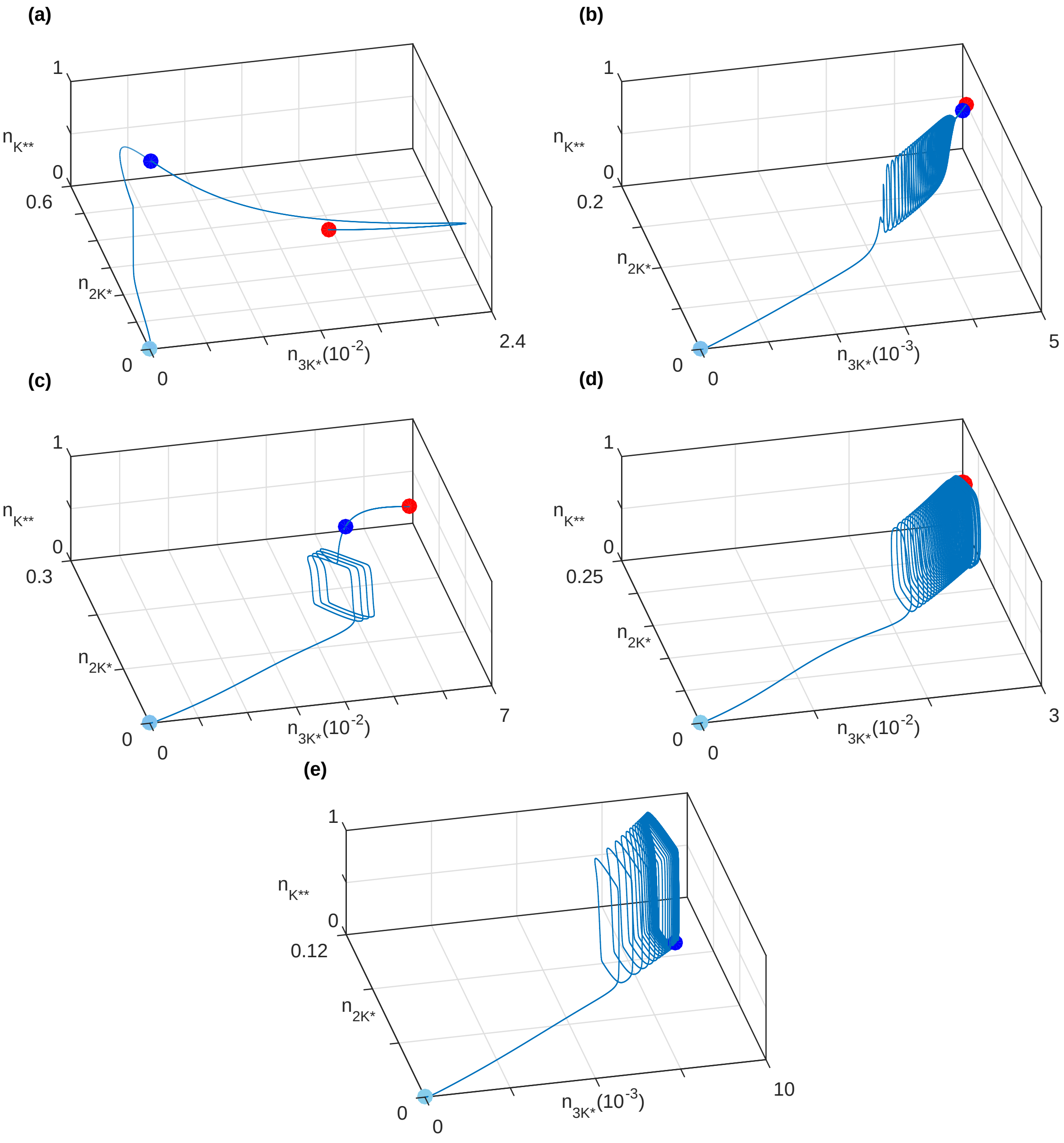

Figure S3: Alternative representation of each of the phase space trajectories shown in Figure 2 (f-j) in the main text. The size and orientation of these panels have been adjusted for clarity.

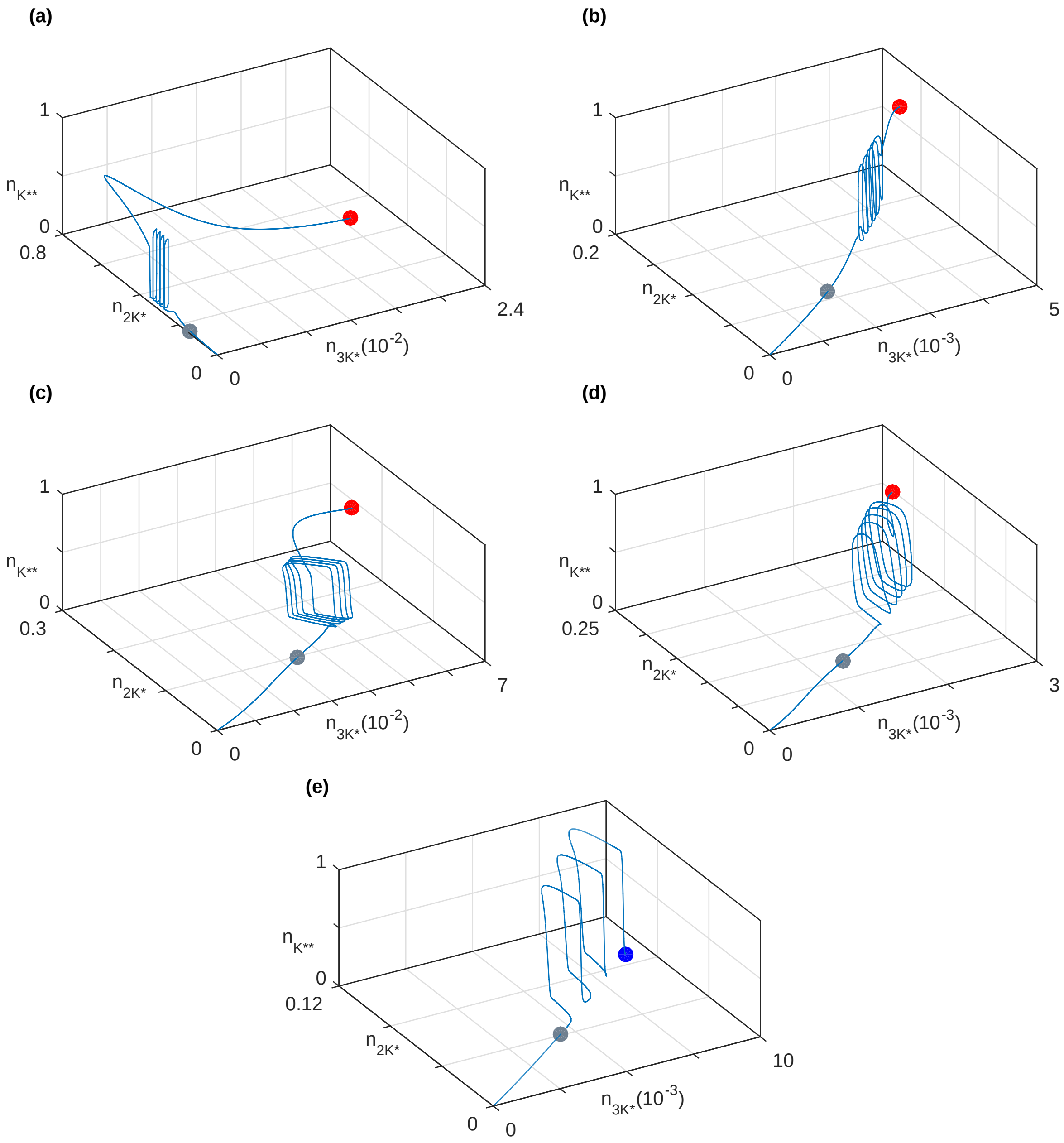

Figure S4: Alternative representation of each of the phase space trajectories shown in Figure 3 (f-j) in the main text. The size and orientation of these panels have been adjusted for clarity.

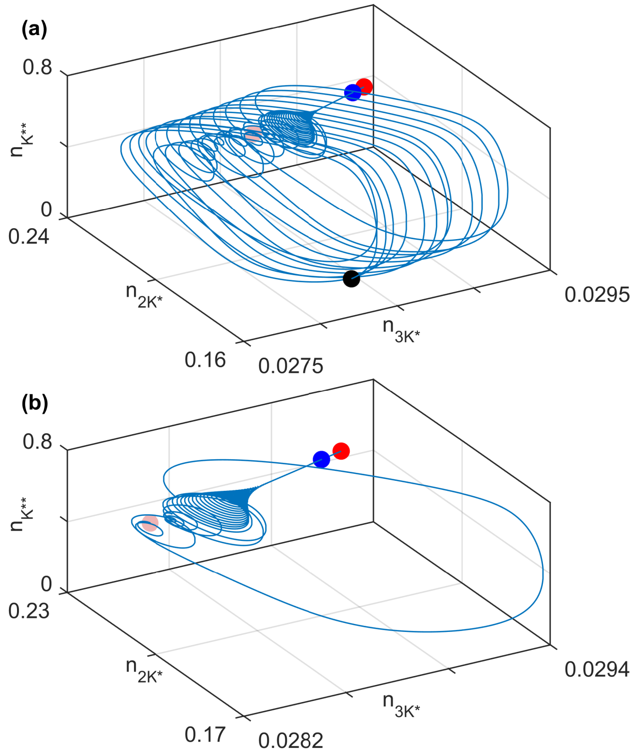

Figure S5: Magnified views of the phase space trajectory shown in Fig. 2(i) in the main text. The blue markers correspond to the final point in the time series displayed in Fig. 2(d), while the red markers indicate the fixed point of the dynamical system in the presence of stimulus. (a) Magnified view of the trajectory beginning from the black marker shown in Fig. 2(i). The pink marker denotes the starting point of the segment of the trajectory displayed in panel (b). (b) Further magnification of a section of the phase-plane trajectory shown in panel (a) corresponding to the duration when the system moves away from the unstable limit cycle and converges to the stable fixed point.

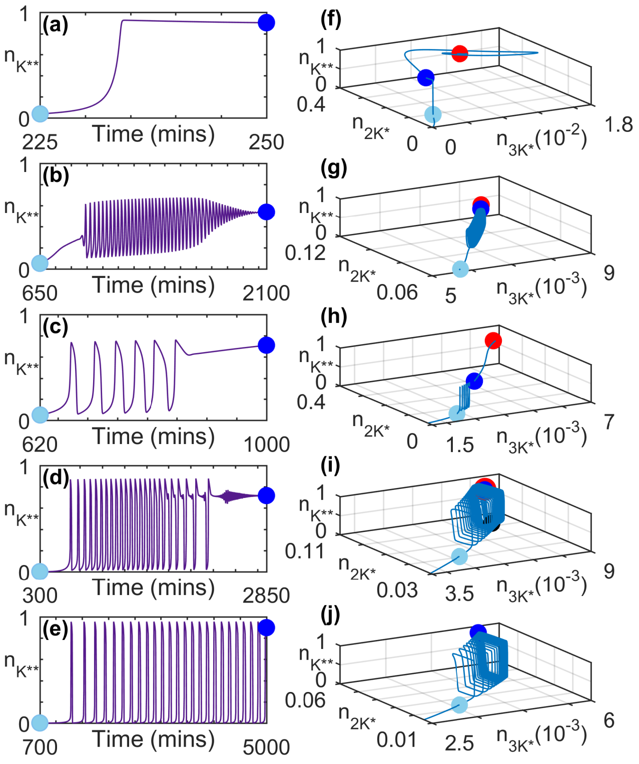

Figure S6: Transient activity in MAPK cascade immediately following the application of a stimulus having amplitude $S = 1.2 \times 10^{-6} \mu M$ at $t = 0$. (a-e) Characteristic time series for the normalized concentration of doubly phosphorylated MAPK ($n_{K^{**}}$) for different total concentrations of kinases (see Table S6. For all panels, the kinetic rates are identical to the MMS set (see Table S2) and phosphatase concentrations are as in Table S5. The concentration of active MAPK is insignificant prior to the time periods shown in panels (a-e). (f-j) Trajectories representing the evolution of the systems in panels (a-e) in the projection of the phase-space on the planes comprising normalized concentrations of active MAP3K (n_{3K^*}), singly phosphorylated MAP2K (n_{2K^*}) and active MAPK ($n_{K^{**}}$). The concentrations have been normalized by the total concentration of MAP3K ($[3K]_{tot}$), MAP2K ($[2K]_{tot}$) and MAPK ($[K]_{tot}$), respectively. The light blue and dark blue markers in each of the panels (f-j) demarcate the portion of the trajectories that correspond to the time series shown in panels (a-e). The steady state of the system is represented by a red marker in panels (f-i). In panels (e) and (j), the system converges to a stable limit cycle.

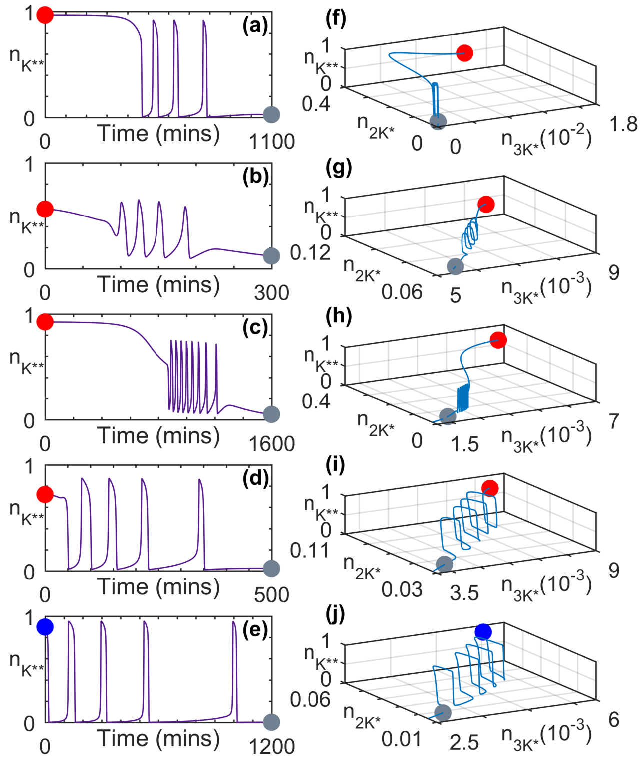

Figure S7: Transient activity in MAPK cascade immediately following the withdrawal (at $t = 0$) of an applied stimulus having amplitude $S = 1.2 \times 10^{-6} \mu M$. (a-e) Characteristic time series for the normalized concentration of doubly phosphorylated MAPK ($n_{K^{**}}$) shown for different total concentrations of kinases (see Table S6). (f-j) Trajectories representing the evolution of the systems in panels (a-e) in the projection of the phase-space on the planes comprising normalized concentrations of active MAP3K (n_{3K^*}), singly phosphorylated MAPK (n_{2K^*}) and active MAPK ($n_{K^{**}}$). The concentrations have been normalized by the total concentration of MAP3K ($[3K]_{tot}$), MAP2K ($[2K]_{tot}$) and MAPK ($[K]_{tot}$), respectively. The steady state of the system prior to the withdrawal of the stimulus is represented by a red marker (panels f-i). The system in panels (e) and (j) is seen to relax from a state characterized by stable limit cycle oscillations (represented by the blue marker). In each trajectory shown in (f-j) the grey marker denotes the state of the system corresponding to the final time point in panels (a-e). The concentration of active MAPK is close to its resting state value following the time period shown in (a-e). The parameter values for each panel are same as those for the corresponding panels in Fig. S6. Note that panel (d) is obtained using the same parameter values as Fig. 4 (d) of the main text.

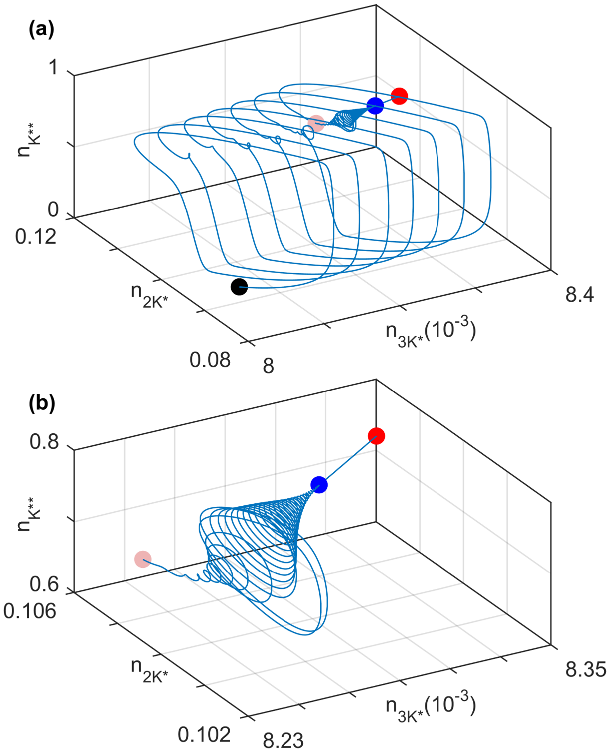

Figure S8: Magnified views of the phase space trajectory shown in Fig. S6 (i). The blue markers correspond to the final point in the time series displayed in Fig. S6 (d), while the red markers indicate the fixed point of the dynamical system in the presence of stimulus. (a) Magnified view of the trajectory beginning from the black marker shown in Fig. S6 (i). The pink marker denotes the starting point of the segment of the trajectory displayed in panel (b). (b) Further magnification of a section of the phase-plane trajectory shown in panel (a) corresponding to the duration when the system moves away from the unstable limit cycle and converges to the stable fixed point.

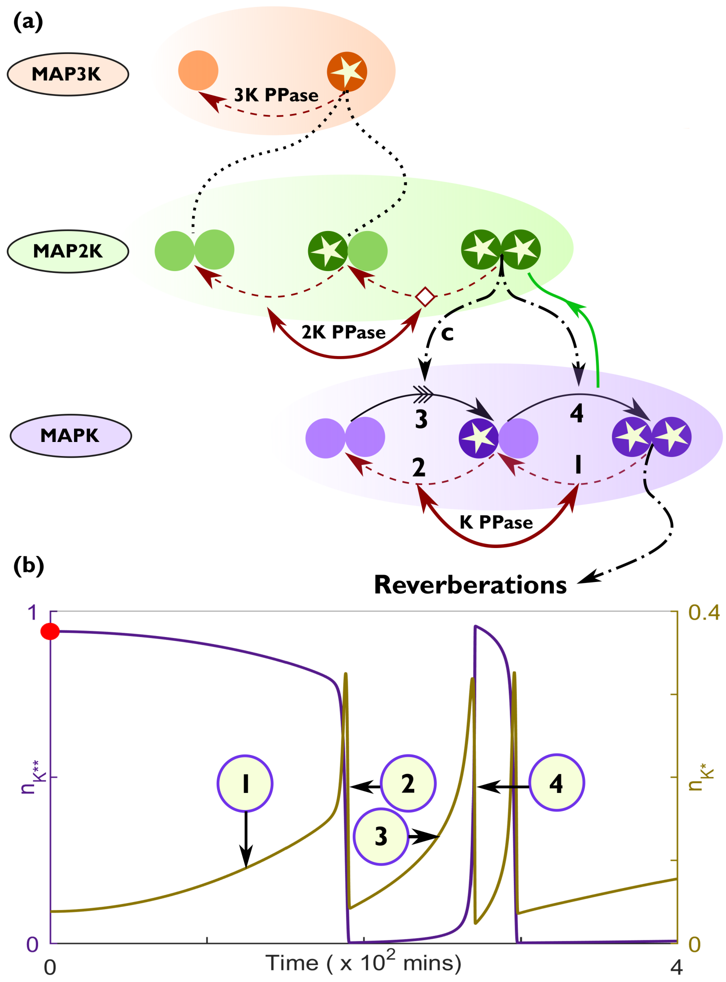

Figure S9: Processes underlying long-lived memory and reverberatory dynamics. (a) Schematic representation of MAPK cascade showing the processes that occur subsequent to removing a stimulus. The numbers $(1-4)$ represent the sequence of events that lead to the emergence of the post-stimulus large-amplitude spiking activity shown in (b). The enzyme-substrate protein complex formed during activation of MAPK by doubly phosphorylated MAP2K is indicated by "c". The green arrow from the MAPK layer to the MAP2K layer represents the release of doubly phosphorylated MAP2K from downstream complexes. (b) A characteristic time-series for the normalized concentration of singly and doubly phosphorylated MAPK (n_{K^*} and $n_{K^{**}}$, respectively) following the removal of an applied stimulus of amplitude $S = 2.0 \times 10^{-6} \mu M$ at $t = 0$. The numbers $(1-4)$ represent the same events shown in (a). The total concentrations of the kinases and phosphatases used for generating the time-series are provided in Tables S4 and S5, respectively.

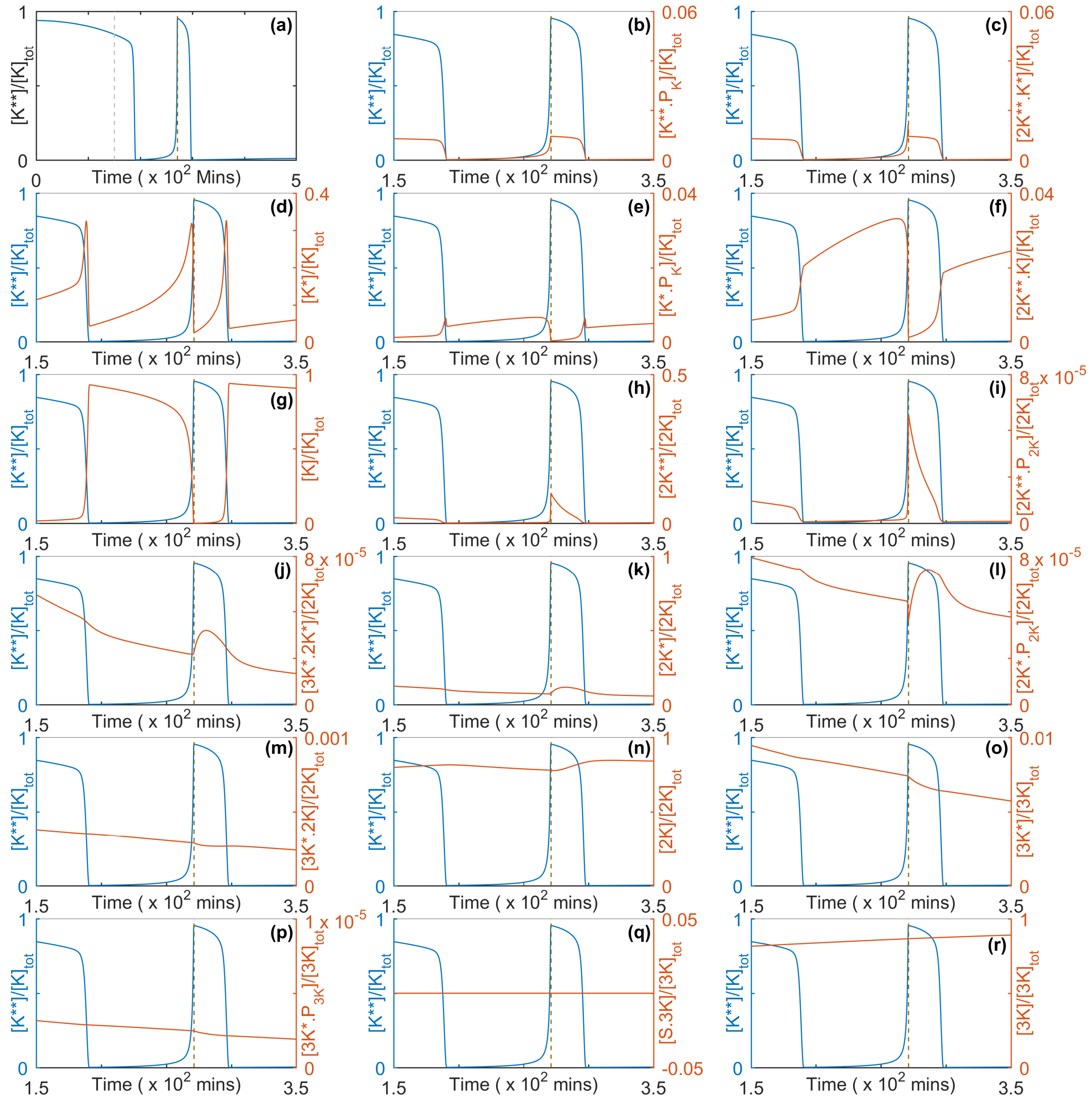

Figure S10: Characteristic dynamics of the molecular components of the MAP Kinase cascade following withdrawal of a stimulus. (a) The time-series of the normalized concentration of doubly phosphorylated MAPK ($[K^{**}]/[K]_{tot}$) following removal of an applied stimulus with amplitude $S = 2.0 \times 10^{-6} \mu M$ at $t = 0$. (b-r) Time-series of the normalized concentrations of the different components of the MAPK cascade, shown starting from $t = 150$ minutes after withdrawing the stimulus, displayed together with the time-series of normalized MAPK activity $[K^{**}]/[K]_{tot}$. The total concentrations of the kinases and phosphatases used for generating the figures are provided in Tables S4 and S5, respectively.

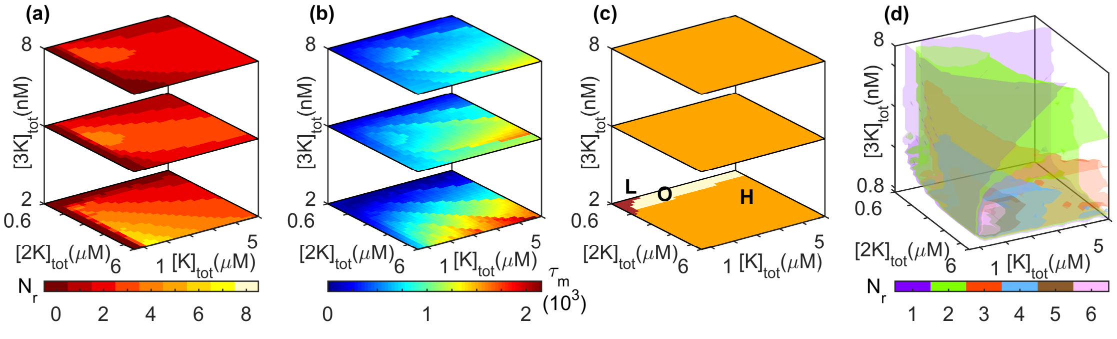

Figure S11: Dependence of the reverberatory activity on the total kinase concentrations, viz., MAPK ($[K]_{tot}$), MAP2K ($[2K]_{tot}$) and MAP3K ($[3K]_{tot}$). The corresponding dynamical attractors of the system under sustained stimulation are also shown. (a) The number of post-stimulus spikes N_r, (b) the total memory time τ_m (in minutes), (c) the corresponding asymptotic dynamical states of the cascade under sustained stimulation, and (d) isosurfaces for N_r observed on withdrawing an applied stimulus of amplitude $S = 2.0 \times 10^{-6} \mu M$, are shown as functions of the three total kinase concentrations. The total concentrations of the phosphatases are held fixed for (a-d) and are provided in Table S5. The kinetic rates used are given in Table S2.

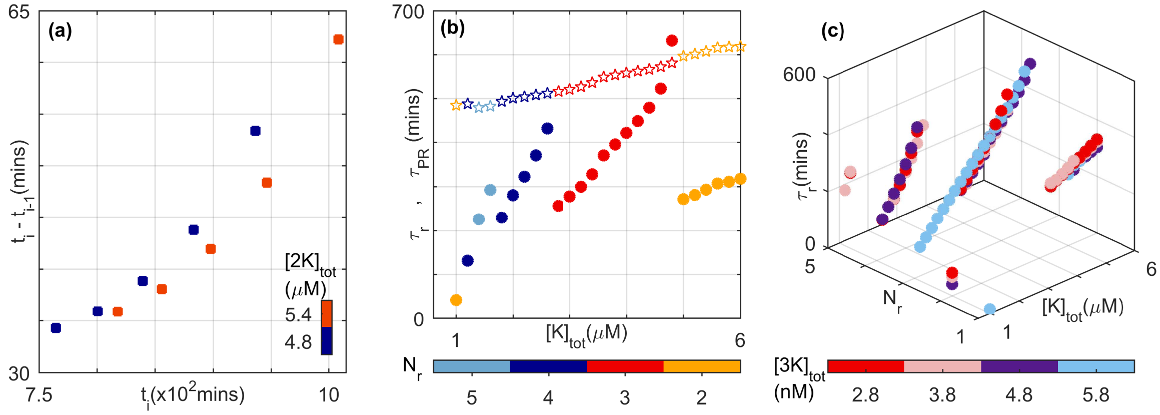

Figure S12: Characterization of the reverberatory dynamics observed after withdrawing a stimulus having amplitude $S = 2.0 \times 10^{-6} \mu M$. (a) The interval between successive spikes $i-1$ and i increases with time (t_i being the time of occurrence of the ith spike) for two distinct total concentrations of MAP2K. The total concentrations of MAPK and MAP3K are $[K]_{tot} = 1.2\mu M$ and $[3K]_{tot} = 2.8nM$, respectively. (b) The primary recovery time $\tau_{\rm PR}$ (stars) and the total duration of reverberatory activity τ_r (filled circles) are shown for different values of N_r (indicated by the color bar). While $\tau_{\rm PR}$ increases monotonically with increasing total MAPK concentration, τ_r shows a more complex dependence ($[2K]_{tot} = 3\mu M$ and $[3K]_{tot} = 4nM$). (c) The dependence of τ_r on $[K]_{tot}$ for different values of N_r has a similar nature for different choices of $[3K]_{tot}$ (indicated by the color bar, $[2K]_{tot} = 3\mu M$). Note that for panel (c), we consider only situations where the system attains a steady state on maintaining the stimulation. For the total concentrations of the phosphatases see Table S5.

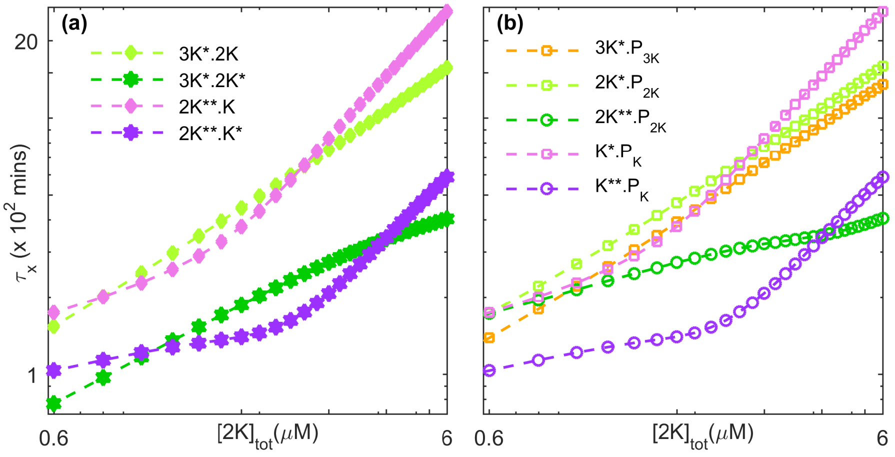

Figure S13: Protein complexes in the MAPK cascade exhibit relaxation behavior occurring over a broad range of time-scales. Decay of activity is shown after withdrawing an applied stimulus of amplitude $S = 1.2 \times 10^{-6} \mu M$. The relaxation times τ_x of the different molecular species, viz., (a) the protein complexes between non-phosphorylated and singly phosphorylated (non-active) kinase proteins and the doubly phosphorylated (active) kinase protein of the preceding layer, and (b) the protein complexes between the phosphorylated (singly- or doubly-) kinase proteins and the phosphatase that carries out dephosphorylation in the corresponding layer of the MAPK cascade, vary with the total concentration of MAP2K. The nature of this dependence is distinct for lower and higher values of $[2K]_{tot}$. For both panels, $[K]_{tot} = 0.8 \mu M$ and $[3K]_{tot} = 0.0020 \mu M$. The total concentrations of the phosphatases are provided in Table S5.

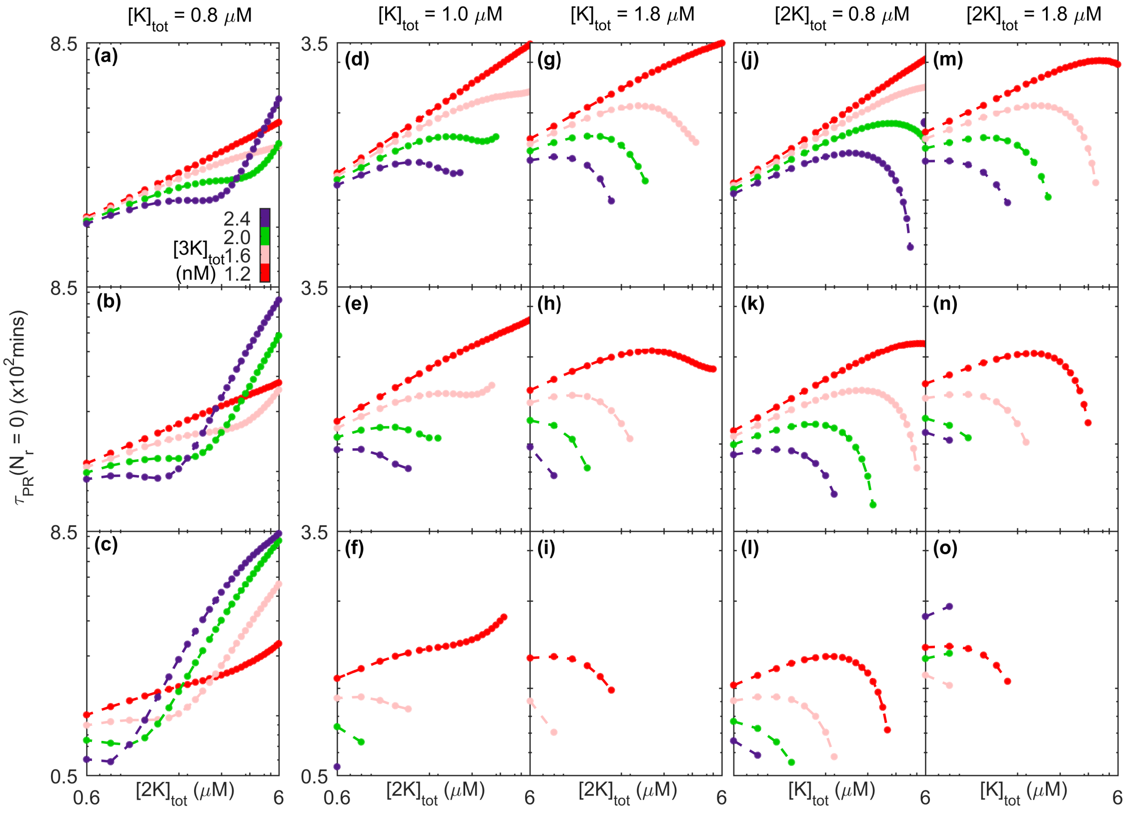

Figure S14: Dependence of the primary recovery time τ_{PR} on (a-i) the total concentration of MAP2K ($[2K]_{tot}$) and on (j-o) the total concentration of MAPK ($[K]_{tot}$) for different values of the total concentration of MAP3K ($[3K]_{tot}$), obtained upon removing stimuli having different amplitudes S. Panels (a,d,g,j,m) are for $S = 0.8 \times 10^{-6}\mu M$, panels (b,e,h,k,n) are for $S = 1.2 \times 10^{-6}\mu M$, and panels (c,f,i,l,o) are for $S = 2.0 \times 10^{-6}\mu M$. We have only considered situations where the system reaches a steady state upon application of a time-invariant stimulus, and that do not show any reverberatory activity ($N_r = 0$) during relaxation to the resting state. The curves in panels (a-i) are obtained for different values of $[K]_{tot}$, namely, (a-c) $[K]_{tot} = 0.8\mu M$, (d-f) $[K]_{tot} = 1.0\mu M$, and (g-i) $[K]_{tot} = 1.8\mu M$. The curves in panels (j-o) are obtained for different values of $[2K]_{tot}$, namely, (j-l) $[2K]_{tot} = 0.8\mu M$, and (m-o) $[2K]_{tot} = 1.8\mu M$. The total concentrations of the phosphatases for all panels are given in Table S5.

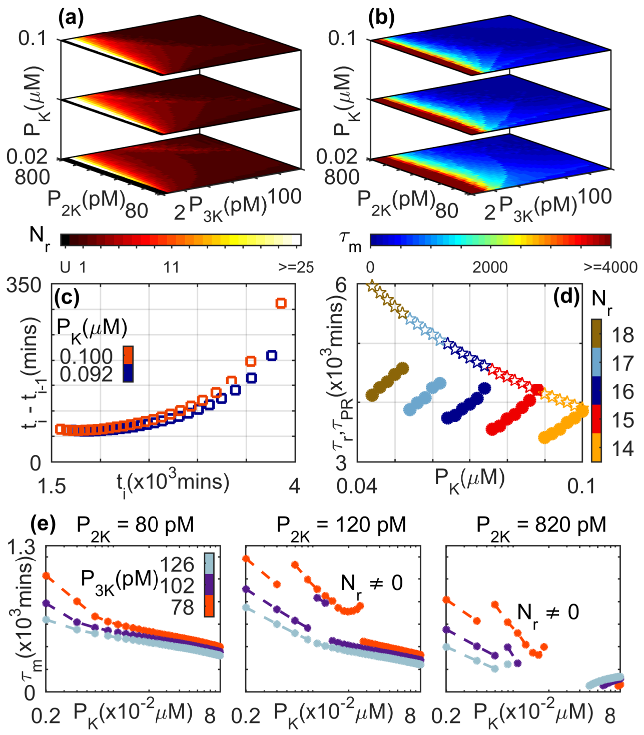

Figure S15: Dependence of reverberatory activity on the total concentrations of the phosphatases MAPK PPase ($[P_K]$), MAP2K PPase, ($[P_{2K}]$) and MAP3K PPase ($[P_{3K}]$). (a) The number of spikes N_r and (b) the total memory time τ_m (in minutes) observed on withdrawing an applied stimulus of amplitude $S = 2.0 \times 10^{-6} \mu M$. Situations where the primary recovery time is longer than a maximum or cut-off value (see Methods), such that the reverberatory nature of the dynamics cannot be properly measured, are indicated by the color corresponding to "U". (c) The interval between successive spikes $i-1$ and i increases with time (t_i being the time of occurrence of the ith spike). As the MAPK PPase concentration is increased, the durations of these intervals are seen to increase. The total concentrations of the other two phosphatases are maintained at $[P_{2K}] = 680pM$ and $[P_{3K}] = 10pM$. (d) The variation of primary recovery time $\tau_{\rm PR}$ (stars) and the total duration of reverberatory activity τ_r (filled circles) as a function of total MAPK PPase concentration are shown for different values of N_r (indicated by the color bar). While $\tau_{\rm PR}$ decreases monotonically with increasing $[P_K]$, τ_r shows a more complex dependence ($[P_{2K}] = 200pM$ and $[P_{3K}] = 6pM$). (e) Dependence of the total memory time τ_m on total MAPK PPase concentration ($[P_K]$ shown in log scale) for different total concentrations of MAP2K PPase (values indicated above each of the three panels) and MAP3K PPase (indicated using different colors as shown in the color bar). Note that we consider only situations where the system attains a steady state on maintaining stimulation. For details of the total concentrations of the kinases, see Table S4.

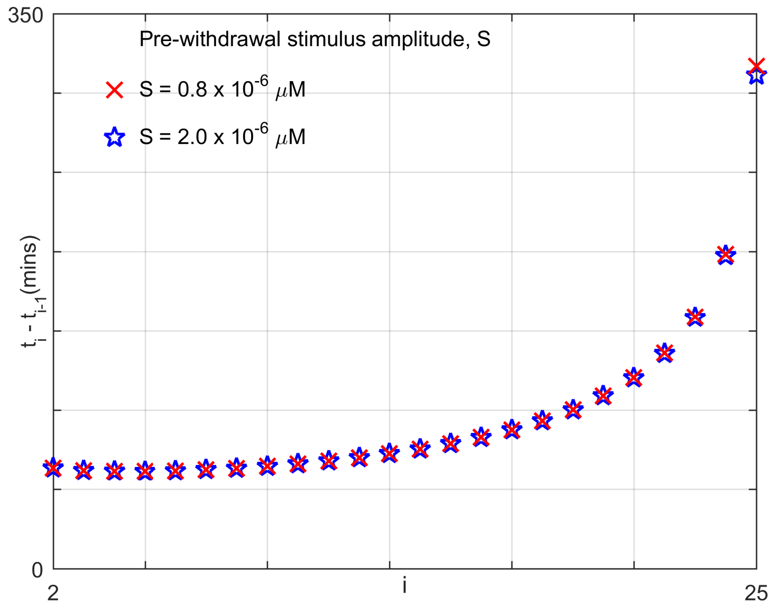

Figure S16: The time interval between successive spikes $i-1$ and i obtained after removing a stimulus, increases with the number of spike events (i being the event number of the ith spike). The trend appears to be independent of the stimulus amplitude S. The total concentrations of the phosphatases are $P_K = 0.1\mu M$, $P_{2K} = 680pM$ and $P_{3K} = 10pM$, respectively. The total concentrations of the kinases are provided in Table S4.

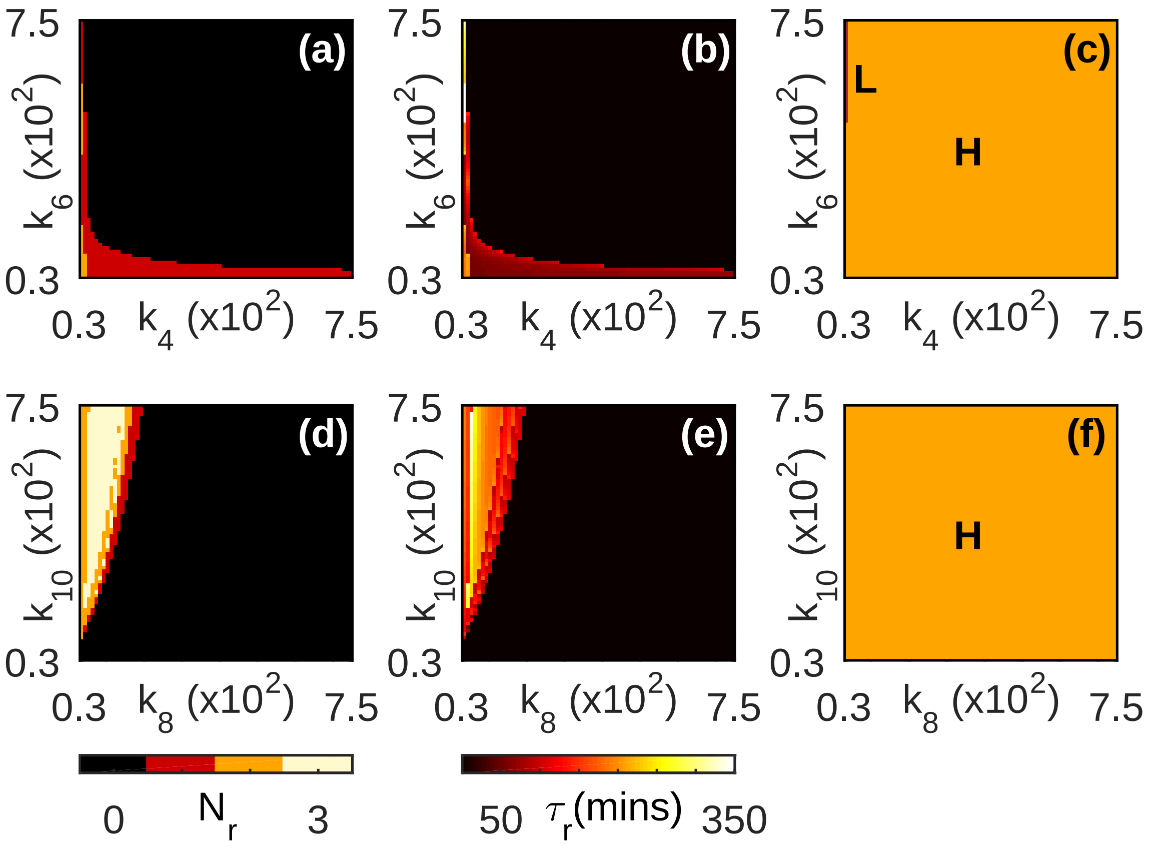

Figure S17: Dependence of reverberatory activity on specific kinetic rates governing the dynamics of the MAPK cascade. The corresponding dynamical attractors of the system under sustained stimulation are also shown. (a,d) The number of post-stimulus spikes N_r and (b,e) the total duration of reverberatory activity τ_r (in minutes) observed on withdrawing the stimulus, as well as, (c,f) the corresponding asymptotic dynamical states of the cascade under sustained stimulation, are shown. They are displayed as a function of the kinetic rates k_4 and k_6 which govern the product formation steps in the single and double phosphorylation reactions (respectively) during MAP2K activation (a-c) and of the kinetic rates k_8 and k_{10} which govern the product formation steps in the single and double phosphorylation reactions (respectively) during MAPK activation (d-f). The rates are expressed in units of min^{-1}. The values of the other reaction rates are chosen to be identical to the reference MMS set (Table S2). The total concentrations of the kinase and phosphatase molecules are same as in Fig. 2(e) in the main text. The strength of the signal used to stimulate the cascade in all cases is $S = 2 \times 10^{-6}$ μM. For comparison note that using the MMS reference set for all parameter values will yield $N_r = 2$, $tau_r = 320.7$ minutes and an asymptotic steady state corresponding to high MAPK activity (H) under sustained stimulation.

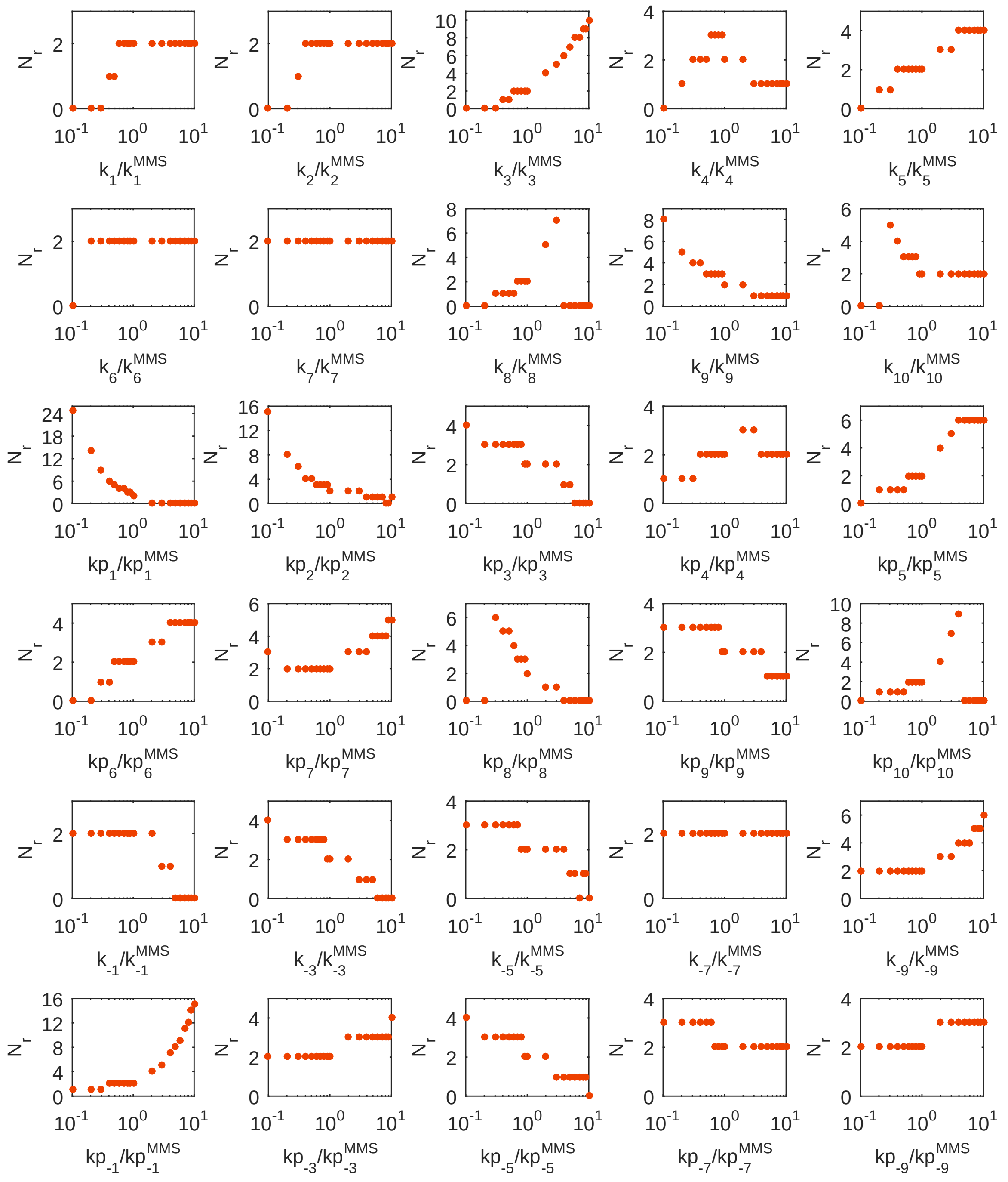

Figure S18: Dependence of the reverberatory activity measured in terms of the number of post-stimulus spikes N_r on each of the kinetic rates governing the MAPK cascade dynamics. In each panel, a specific kinetic rate is varied ten-fold either way from the corresponding MMS reference value while keeping all other parameters fixed at the respective value in the MMS reference set (Table S2). The total concentrations of the kinase and phosphatase molecules are same as in Fig. 2(e) in the main text. The strength of the signal used to stimulate the cascade in all cases is $S = 2 \times 10^{-6}\ \mu M$. For comparison note that using the MMS reference set for all parameter values will yield $N_r = 2$.

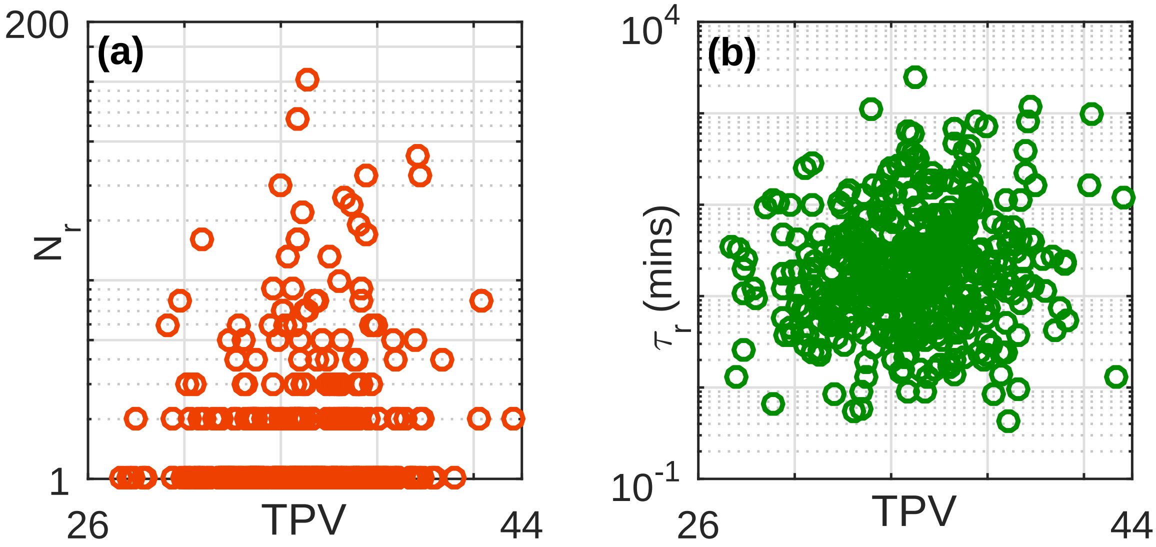

Figure S19: Robustness of the observed reverberatory activity in MAPK cascade following withdrawal of applied stimulus having strength $S = 5 \times 10^{-6} \mu M$ with respect to variation in the system parameters. The panels show (a) the number of spikes during relaxation N_r and (b) the total duration of reverberatory activity τ_r, on the Total Parameter Variation (TPV) as defined in the main text (see Methods). The circles in each panel represent an individual realization of the cascade dynamics where each parameter set is chosen by uniform random sampling from a physiologically plausible range (see Table S3).
